# Strains used in whole organism *Plasmodium falciparum* vaccine trials differ in genome structure, sequence, and immunogenic potential

**DOI:** 10.1186/s13073-019-0708-9

**Published:** 2020-01-08

**Authors:** Kara A. Moser, Elliott F. Drábek, Ankit Dwivedi, Emily M. Stucke, Jonathan Crabtree, Antoine Dara, Zalak Shah, Matthew Adams, Tao Li, Priscila T. Rodrigues, Sergey Koren, Adam M. Phillippy, James B. Munro, Amed Ouattara, Benjamin C. Sparklin, Julie C. Dunning Hotopp, Kirsten E. Lyke, Lisa Sadzewicz, Luke J. Tallon, Michele D. Spring, Krisada Jongsakul, Chanthap Lon, David L. Saunders, Marcelo U. Ferreira, Myaing M. Nyunt, Miriam K. Laufer, Mark A. Travassos, Robert W. Sauerwein, Shannon Takala-Harrison, Claire M. Fraser, B. Kim Lee Sim, Stephen L. Hoffman, Christopher V. Plowe, Joana C. Silva

**Affiliations:** 1 Institute for Genome Sciences, University of Maryland School of Medicine, Baltimore, MD 21201 USA; 2 Present address: Institute for Global Health and Infectious Diseases, University of North Carolina Chapel Hill, Chapel Hill, USA; 3 Center for Vaccine Development and Global Health, University of Maryland School of Medicine, Baltimore, MD 21201 USA; 4 Sanaria, Inc., Rockville, MD 20850 USA; 5 Department of Parasitology, Institute of Biomedical Sciences, University of São Paulo, São Paulo, Brazil; 6 Genome Informatics Section, Computational and Statistical Genomics Branch, National Human Genome Research Institute, Bethesda, MD 20892 USA; 7 Department of Bacterial and Parasitic Diseases, Armed Forces Research Institute of Medical Sciences, Bangkok, Thailand; 8 Present address: Warfighter Expeditionary Medicine and Treatment, US Army Medical Material Development Activity, Frederick, USA; 9 Present address: Duke Global Health Institute, Duke University, Durham, NC 27708 USA; 10 Department of Medical Microbiology, Radboud University Medical Center, Nijmegen, Netherlands; 11 Department of Microbiology and Immunology, University of Maryland School of Medicine, Baltimore, MD 21201 USA

**Keywords:** *P. falciparum*, Malaria, Genome assembly, PfSPZ vaccine, Whole-sporozoite vaccine

## Abstract

**Background:**

*Plasmodium falciparum* (Pf) whole-organism sporozoite vaccines have been shown to provide significant protection against controlled human malaria infection (CHMI) in clinical trials. Initial CHMI studies showed significantly higher durable protection against homologous than heterologous strains, suggesting the presence of strain-specific vaccine-induced protection. However, interpretation of these results and understanding of their relevance to vaccine efficacy have been hampered by the lack of knowledge on genetic differences between vaccine and CHMI strains, and how these strains are related to parasites in malaria endemic regions.

**Methods:**

Whole genome sequencing using long-read (Pacific Biosciences) and short-read (Illumina) sequencing platforms was conducted to generate de novo genome assemblies for the vaccine strain, NF54, and for strains used in heterologous CHMI (7G8 from Brazil, NF166.C8 from Guinea, and NF135.C10 from Cambodia). The assemblies were used to characterize sequences in each strain relative to the reference 3D7 (a clone of NF54) genome. Strains were compared to each other and to a collection of clinical isolates (sequenced as part of this study or from public repositories) from South America, sub-Saharan Africa, and Southeast Asia.

**Results:**

While few variants were detected between 3D7 and NF54, we identified tens of thousands of variants between NF54 and the three heterologous strains. These variants include SNPs, indels, and small structural variants that fall in regulatory and immunologically important regions, including transcription factors (such as PfAP2-L and PfAP2-G) and pre-erythrocytic antigens that may be key for sporozoite vaccine-induced protection. Additionally, these variants directly contributed to diversity in immunologically important regions of the genomes as detected through in silico CD8^+^ T cell epitope predictions. Of all heterologous strains, NF135.C10 had the highest number of unique predicted epitope sequences when compared to NF54. Comparison to global clinical isolates revealed that these four strains are representative of their geographic origin despite long-term culture adaptation; of note, NF135.C10 is from an admixed population, and not part of recently formed subpopulations resistant to artemisinin-based therapies present in the Greater Mekong Sub-region.

**Conclusions:**

These results will assist in the interpretation of vaccine efficacy of whole-organism vaccines against homologous and heterologous CHMI.

**Electronic supplementary material:**

The online version of this article (10.1186/s13073-019-0708-9) contains supplementary material, which is available to authorized users.

## Background

The flattening levels of mortality and morbidity due to malaria in recent years [1], which follow a decade in which malaria mortality was cut in half, highlight the pressing need for new tools to control this disease. A highly efficacious vaccine against *Plasmodium falciparum*, the deadliest malaria parasite, would be a critical development for control and elimination efforts. Several variations of a highly promising pre-erythrocytic, whole organism malaria vaccine based on *P. falciparum* sporozoites (PfSPZ) are under development, all based on the same *P. falciparum* strain, NF54 [2], thought to be of West African origin, and which use different mechanisms for attenuation of PfSPZ. Of these vaccine candidates, Sanaria® PfSPZ vaccine, based on radiation-attenuated sporozoites, has progressed furthest in clinical trial testing [3–9]. Other whole-organism vaccine candidates, including chemoattenuated (Sanaria® PfSPZ-CVac), transgenic, and genetically attenuated sporozoites, are in earlier stages of development [10–12].

PfSPZ vaccine showed 100% short-term protection against homologous controlled human malaria infection (CHMI) in an initial phase 1 clinical trial [5], and subsequent trials have confirmed that high levels of protection can be achieved against both short-term [7] and long-term [6] homologous CHMI. However, depending on the immunization regimen, sterile protection can be significantly lower (8–83%) against heterologous CHMI using the 7G8 Brazilian clone [7, 8], and against infection in malaria-endemic regions with intense seasonal malaria transmission (29% and 52% by proportional and time to event analysis, respectively) [9]. Heterologous CHMI in chemoprophylaxis with sporozoites trials, in which immunization is by infected mosquito bite of individuals undergoing malaria chemoprophylaxis, have been conducted with NF135.C10 from Cambodia [13] and NF166.C8 from Guinea [14], and have had lower efficacy than against homologous CHMI [15, 16]. One explanation for the lower efficacy seen against heterologous *P. falciparum* strains is the extensive genetic diversity in this parasite species, which is particularly high in genes encoding antigens [17] and which combined with low vaccine efficacy against non-vaccine alleles [18–20] reduces overall protective efficacy and complicates the design of broadly efficacious vaccines [21, 22]. The lack of a detailed genomic characterization of the *P. falciparum* strains used in CHMI studies and the unknown genetic basis of the parasite targets of PfSPZ vaccine- and PfSPZ CVac-induced protection have precluded a conclusive statement regarding the cause(s) of variable vaccine efficacy outcomes.

The current PfSPZ vaccine strain, NF54, was isolated from a patient in the Netherlands who had never left the country and is considered a case of “airport malaria;” the exact origin of NF54 is unknown [2], but is thought to be from Africa [23, 24]. NF54 is also the isolate from which the *P. falciparum* 3D7 reference strain was cloned [25], and hence, despite having been separated in culture for over 30 years, NF54 and 3D7 are assumed to be genetically identical, and 3D7 is often used in homologous CHMI [5, 7]. Several issues hinder the interpretation of both homologous and heterologous CHMI experiments conducted to date. It remains to be confirmed that 3D7 has remained genetically identical to NF54 genome-wide, or that the two are at least identical immunogenically. Indeed, NF54 and 3D7 have several reported phenotypic differences when grown in culture, including the variable ability to produce gametocytes [26]. In addition, 7G8, NF166.C8, and NF135.C10 have not been rigorously compared to each other or to NF54 to confirm that they are adequate heterologous strains, even though they do appear to have distinct infectivity phenotypes when used as CHMI strains [14, 16]. While the entire sporozoite likely offers multiple immunological targets, no high-confidence correlates of protection currently exist. In part because of the difficulty of studying hepatic parasite forms and their gene expression profiles in humans, it remains unclear which parasite proteins are recognized by the human immune system during that stage, and elicit protection, upon immunization with PfSPZ vaccines. Both humoral and cell-mediated responses have been associated with protection against homologous CHMI [5, 6], although studies in rodents and non-human primates point to a requirement for cell-mediated immunity (specifically through tissue-resident CD8^+^ T cells) in long-term protection [4, 8, 27, 28]. In silico identification of CD8^+^ T cell epitopes in all strains could highlight critical differences of immunological significance between strains. Finally, heterologous CHMI results cannot be a reliable indicator of efficacy against infection in field settings unless the CHMI strains used are characteristic of the geographic region from which they originate. These issues could impact the use of homologous and heterologous CHMI, and the choice of strains for these studies, to predict the efficacy of PfSPZ-based vaccines in the field [29].

These knowledge gaps can be addressed through a rigorous description and comparison of the genome sequence of these strains. High-quality de novo assemblies allow characterization of genome composition and structure, as well as the identification of genetic differences between strains. However, the high AT content and repetitive nature of the *P. falciparum* genome greatly complicates genome assembly methods [30]. Recently, long-read sequencing technologies have been used to overcome some of these assembly challenges, as was shown with assemblies for 3D7, 7G8, and several other culture-adapted *P. falciparum* strains generated using Pacific Biosciences (PacBio) technology [31–33]. However, NF166.C8 and NF135.C10 still lack whole-genome assemblies; in addition, while an assembly for 7G8 is available [32], it is important to characterize the specific 7G8 clone used in heterologous CHMI, from Sanaria’s working bank, as strains can undergo genetic changes over time in culture [34]. Here, reference assemblies for NF54, 7G8, NF166.C8, and NF135.C10 (hereafter referred to as PfSPZ strains) were generated using approaches to take advantage of the resolution power of long-read sequencing data and the low error rate of short-read sequencing platforms. These de novo assemblies allowed for the thorough genetic and genomic characterization of the PfSPZ strains and will aid in the interpretation of results from CHMI studies.

## Methods

### Study design and samples

This study characterized and compared the genomes of four *P. falciparum* strains used in whole organism malaria vaccines and controlled human malaria infections using a combination of long- and short-read whole genome sequencing platforms (see below). In addition, these strains were compared to *P. falciparum* clinical isolates collected from patients in malaria-endemic regions globally, using short-read whole genome sequencing data. Genetic material for the four PfSPZ strains were provided by Sanaria, Inc. Clinical *P. falciparum* isolates from Brazil, Mali, Malawi, Myanmar, and Thailand were collected between 2009 and 2016 from cross-sectional surveys of malaria burden, longitudinal studies of malaria incidence, and drug efficacy studies done in collaboration with the Malaria Research Program within the Center for Vaccine Development and Global Health at the University of Maryland, Baltimore, or were otherwise provided by collaborators (Additional file 1). All samples met the inclusion criteria of the initial study protocol with prior approval from the local ethical review board. Parasite genomic sequencing and analyses were undertaken after approval of the University Of Maryland School Of Medicine Institutional Review Board was received. These isolates were obtained by venous blood draws; almost all samples were processed using leukocyte depletion methods to improve the parasite-to-human DNA ratio before sequencing. The exceptions were samples from Brazil and Malawi, which were not leukocyte depleted upon collection. These samples underwent a selective whole genome amplification step before sequencing, modified from [35] (the main modification being a DNA dilution and filtration step using vacuum filtration prior to selective whole genome amplification [36]). Additionally, samples for which whole genome short-read sequencing was previously generated were obtained from NCBI’s Short Read Archive to supplement the following malaria-endemic regions not represented in our data set and regions where PfSPZ trials are ongoing [37–39]: Peru, Columbia, French Guiana, Guinea, Cambodia, Papua New Guinea, Burkina Faso, Kenya, and Tanzania (Additional file 1).

### Whole genome sequencing

Genetic material for whole genome sequencing of the PfSPZ strains was generated from a cryovial of each strain’s cell bank with the following identifiers: NF54 Working Cell Bank (WCB): SAN02-073009; 7G8 WCB: SAN02-021214; NF135.C10 WCB: SAN07-010410; NF166.C8 Mother Cell Bank: SAN30-020613. Each cryovial was thawed and maintained in human O+ red blood cells (RBCs), from Vitalant (Blood System, Inc.), Phoenix, AZ, at 2% hematocrit (Hct) in complete growth medium (RPMI 1649 with l-glutamine and 25 mM HEPES supplemented with 10% human O+ serum and hypoxanthine) in a six-well plate in 5% O_2_, 5% CO_2_, and 90% N_2_ at 37 °C. The cultures were then further expanded by adding fresh RBCs every 3–4 days and increased culture hematocrit (Hct) to 5% Hct using a standard method [40]. The complete growth medium was replaced daily. When the PfSPZ strain culture volume reached 300–400 mL and a parasitemia of more than 1.5%, the culture suspensions were collected and the parasitized RBCs were pelleted down by centrifugation at 1800 rpm for 5 min. Aliquots of 0.5 mL per cryovial of the parasitized RBCs were stored at − 80 °C prior to extraction of genomic DNA. Genomic DNA was extracted using the Qiagan Blood DNA Midi Kit (Valencia, CA, USA). Pacific Biosciences (PacBio) sequencing was done for each PfSPZ strain. Total DNA was prepared for PacBio sequencing using the DNA Template Prep Kit 2.0 (Pacific Biosciences, Menlo Park, CA). DNA was fragmented with the Covaris E210, and the fragments were size selected to include those > 15 kbp in length. Libraries were prepared per the manufacturer’s protocol. Four SMRT cells were sequenced per library, using P6C4 chemistry and a 120-min movie on the PacBio RS II (Pacific Biosystems, Menlo Park, CA).

Short-read sequencing was done for each PfSPZ strain and for our collection of clinical isolates using the Illumina HiSeq 2500 or 4000 platforms. Prepared genomic DNA, extracted from cultured parasites, leukocyte-depleted samples, or from samples that underwent sWGA (see above), was used to construct DNA libraries for sequencing on the Illumina platform using the KAPA Library Preparation Kit (Kapa Biosystems, Woburn, MA). DNA was fragmented with the Covaris E210 or E220 to ~ 200 bp. Libraries were prepared using a modified version of the manufacturer’s protocol. The DNA was purified between enzymatic reactions and the size selection of the library was performed with AMPure XT beads (Beckman Coulter Genomics, Danvers, MA). When necessary, a PCR amplification step was performed with primers containing an index sequence of six nucleotides in length. Libraries were assessed for concentration and fragment size using the DNA High Sensitivity Assay on the LabChip GX (Perkin Elmer, Waltham, MA). Library concentrations were also assessed by qPCR using the KAPA Library Quantification Kit (Complete, Universal) (Kapa Biosystems, Woburn, MA). The libraries were pooled and sequenced on a 100–150 -bp paired-end Illumina HiSeq 2500 or 4000 run (Illumina, San Diego, CA).

### Assembly generation and characterization of PfSPZ strains

Canu (v1.3) [41] was used to correct and assemble the PacBio reads (corMaxEvidenceErate = 0.15 for AT-rich genomes, default parameters otherwise). Organelle genomes were circularized using Circlator (default settings, accessed October 2019) [42]. To optimize downstream assembly correction processes and parameters, the percentage of total differences (both in bp and by proportion of the 3D7 genome not captured by the NF54 assembly) between the NF54 assembly and the 3D7 reference (PlasmoDBv24) was calculated after each round of correction. Quiver (smrtanalysis v2.3) [43] was run iteratively with default parameters to reach a (stable) maximum reduction in percent differences between the two genomes and the assemblies were further corrected with Illumina data using Pilon (v1.13) [44] with the following parameters: --fixbases, --mindepth 5, --K 85, --minmq 0, and --minqual 35. The 3D7 annotation was mapped onto each assembly using gmap [45] (2014-06-10 version) the following settings: -Y -B 5 -t 10 -K 1500 --cross-species.

Assemblies were compared to the 3D7 reference (PlasmoDBv24) using MUMmer’s nucmer [46], and the show-snps function was used to generate a list of SNPs and small (< 50 bp) indels between assemblies. Coding and non-coding variants were classified by comparing the show-snps output with the 3D7 gff3 file using custom scripts. For a subset of genes which are discussed specifically below (transcription factors, confirmed or suspected pre-erythrocytic genes, variants detected in NF54 relative to 3D7, etc.), small variants were confirmed through manual inspection of extracted (using annotation coordinates) sequence alignments using clustal omega [47]. Structural variants, defined as indels, deletions, and tandem or repeat expansion and contractions each greater than 50 bp in length were identified using the nucmer-based Assemblytics tool [48] (unique anchor length: 1 kbp). Translocations were identified by eye through inspection of mummerplots and confirmed through independent assembly runs using different assemblers and data generated with different sequencing technologies (see Additional file 2: Supplemental Text).

Reconstructed exon 1 sequences for *var* genes, encoding *P. falciparum* erythrocyte membrane protein 1 (PfEMP1) antigens, for each PfSPZ strain were recovered using the ETHA package [49]. As a check for *var* exon 1 sequences that were missed during the generation of the strain’s assembly, a targeted read capture and assembly approach was done using a strain’s Illumina data, wherein *var*-like reads for each PfSPZ strain were identified by mapping reads against a database of known *var* exon 1 sequences [50] using bowtie2 [51]. Reads that mapped to a known exon 1 sequence plus their mate pairs were then assembled with Spades (v3.9.0) [52], and the assembled products were blasted against the PacBio reads to determine if they were exon 1 sequences missed by the de novo assembly process, or if instead they were chimeras reconstructed by the targeted assembly process. To describe *var* sequences in the three heterologous CHMI strains, exon 1 sequences longer than 2.5 kb in length were kept for further characterization. Domain composition was determined using VarDom v1 [50]. Categorization of upstream promoter (UPS) classification, and identification of domain cassette 8/13 *var*s, was done using HMMER [53], using profiles built from known sequences of UPSA-E, DBLα, and CIDRα [50]. (UPS classification was not possible for a small number of sequences found within 10 kb of the end of a contig, or for fragmented sequences).

### In silico MHC I epitope predictions

Given the reported importance of CD8^+^ T cell responses towards immunity to whole sporozoites, MHC class I epitopes of length 9 amino acids were predicted with NetMHCpan (v3.0) [54] for each PfSPZ strain using protein sequences of 42 pre-erythrocytic genes of interest. Likely involvement in pre-erythrocytic immunity was inferred either from a literature review or experimentally, i.e., genes whose products were recognized by sera from protected vaccinees participating in whole organism malaria vaccine trials (both PfSPZ and PfSPZ-CVac) (*n* = 42) [10, 55]. (While the latter were detected through antibody responses, many have also been shown to have T cell epitopes, such as circumsporozoite protein and liver stage antigen 1). HLA types common to African countries where PfSPZ or PfSPZ-CVac trials are ongoing were used for epitope predictions based on frequencies in the Allele Frequency Net Database [56] or from the literature [57, 58] (Additional file 2: Table S1). Shared epitopes between NF54 and the three heterologous PfSPZ strains were calculated by first identifying epitopes in each gene, and then removing duplicate epitope sequence entries (caused by recognition by multiple HLA types). Identical epitope sequences that were identified in two or more genes were treated as distinct epitope entries, and all unique “epitope-given-gene” combinations were included when calculating the number of shared epitopes between strains. To validate these in silico predictions, the predicted epitopes were compared to a published database of experimentally validated CD8^+^ T cell epitopes (filtered to remove epitope sequences longer than 20 amino acids in length) [59].

### Read mapping and SNP calling

For the full collection of clinical isolates that had whole genome short-read sequencing data (generated either at IGS or downloaded from SRA), reads were aligned to the 3D7 reference genome (PlasmoDBv24) using bowtie2 (v2.2.4) [51]. Samples with less than 10 million reads mapping to the reference were excluded, as samples with less than this amount had reduced coverage across the genome. Bam files were processed according to GATK’s Best Practices documentation [60–62]. Joint SNP calling was done using Haplotype Caller (v4.0). Because clinical samples may be polyclonal (that is, more than one parasite strain may be present), diploid calls were initially allowed, followed by calling the major allele at positions with heterozygous calls. If the major allele was supported by > 70% of reads at a heterozygous position, the major allele was assigned as the allele at that position (otherwise, the genotype was coded as missing). Additional hard filtering was done to remove potential false positives based on the following filter: DP < 12 || QUAL < 50 || FS > 14.5 || MQ < 20. Variants were further filtered to remove those for which the non-reference allele was not present in at least three samples (frequency less than ~ 0.5%), and those with more than 10% missing genotype values across all samples.

### Principal coordinate analyses and admixture analyses

A matrix of pairwise genetic distances was constructed from biallelic non-synonymous SNPs identified from the above pipeline (*n* = 31,761) across all samples (*n* = 654) using a custom Python script, and principal coordinate analyses (PCoAs) were done to explore population structure using cmdscale in R. Additional population structure analyses were done using Admixture (v1.3) [63] on two separate data sets: South America and Africa clinical isolates plus NF54, NF166.C8, and 7G8 (*n* = 461), and Southeast Asia and Oceania plus NF135.C10 (*n* = 193). The data sets were additionally pruned for sites in linkage disequilibrium (window size of 20 kbp, window step of 2 kbp, *R*^2^ ≥ 0.1). The final South America/Africa and Southeast Asia/Oceania data set used for the admixture analysis consisted of 16,802 and 5856 SNPs, respectively. The number of populations, *K*, was tested for values between *K* = 1 to *K* = 15 and run with 10 replicates for each *K*. For each population, the cross-validation (CV) error from the replicate with the highest log-likelihood value was plotted, and the *K* with the lowest CV value was chosen as the final *K*.

To compare subpopulations identified in our Southeast Asia/Oceania admixture analysis with previously described ancestral, resistant, and admixed subpopulations from Cambodia [64], the above non-synonymous SNP set was used before pruning for LD (*n* = 11,943) and was compared to a non-synonymous SNP dataset (*n* = 21,257) from 167 samples used by Dwivedi et al. [65] to describe eight Cambodian subpopulations, in an analysis that included a subset of samples used by Miotto et al. [64] (who first characterized the population structure in Cambodia). There were 5881 shared non-synonymous SNPs between the two datasets, 1649 of which were observed in NF135.C10. A pairwise genetic distance matrix (estimated as the proportion of base-pair differences between pairs of samples, not including missing genotypes) was generated from the 5881 shared SNP set, and a dendrogram was built using Ward minimum variance methods in R (Ward.D2 option of the hclust function).

## Results

### Generation of assemblies

**Fig. 1:**
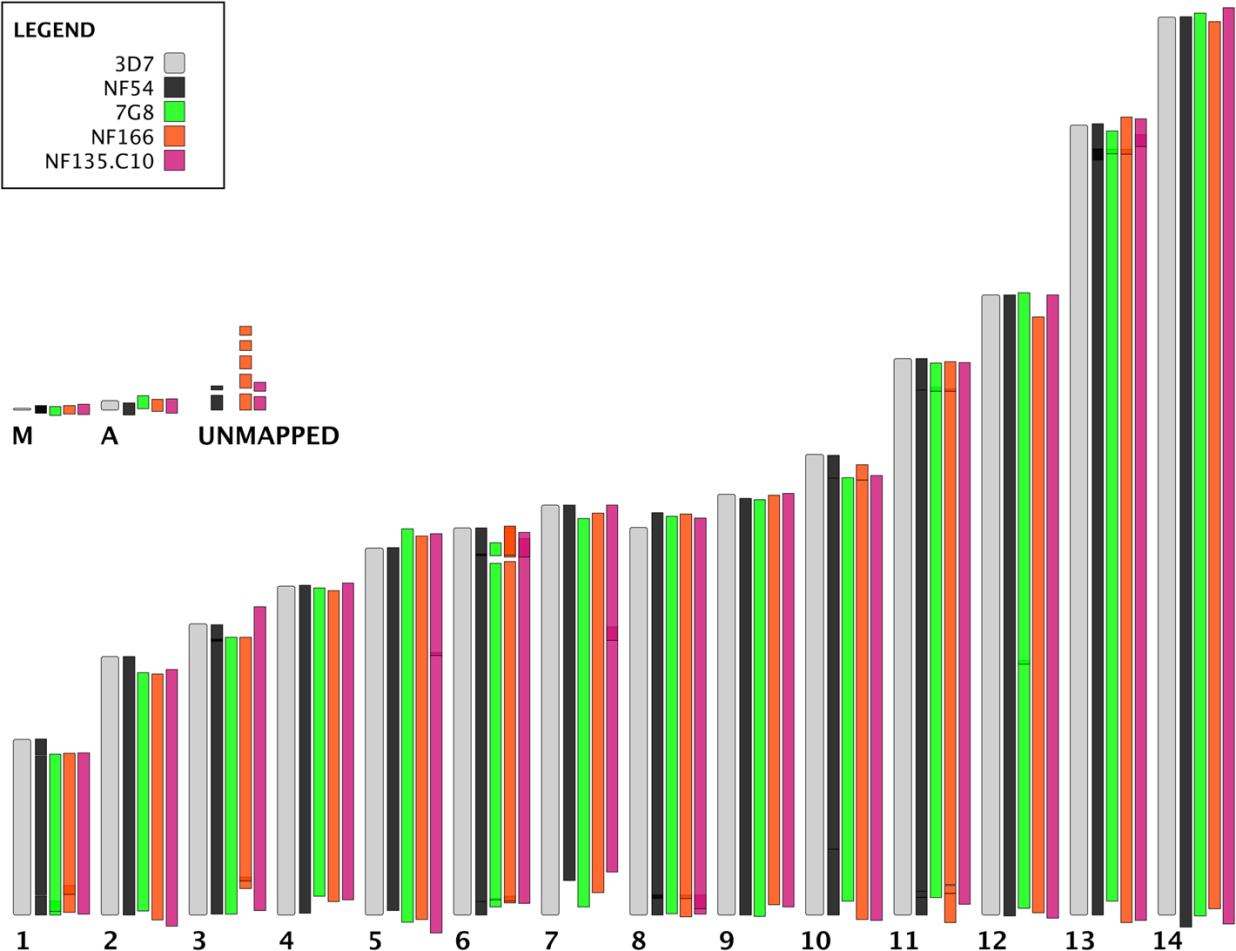
PacBio Assemblies for each PfSPZ strain reconstruct entire chromosomes in one to three continuous pieces. To determine the likely position of each non-reference contig on the 3D7 reference genome, MUMmer’s show-tiling program was used with relaxed settings (-g 100000 -v 50 -i 50) to align contigs to 3D7 chromosomes (top). 3D7 nuclear chromosomes [1–14] are shown in gray, arranged from smallest to largest, along with organelle genomes (M = mitochondrion, A = apicoplast). Contigs from each PfSPZ assembly (NF54: black, 7G8: green, NF166.C8: orange, NF135.C10: hot pink) are shown aligned to their best 3D7 match. A small number of contigs could not be unambiguously mapped to the 3D7 reference genome (unmapped)

To characterize genome-wide structural and genetic diversity of the PfSPZ strains, genome assemblies were generated de novo using whole genome long-read (PacBio) and short-read (Illumina) sequence data (“Methods”; Additional file 2: Table S2 &Table S3). Taking advantage of the parent isolate-clone relationship between NF54 and 3D7, we used NF54 as a test case to derive the assembly protocol, by adopting, at each step, approaches that minimized the difference to 3D7 (Additional file 2: Supplemental Text & Figure S1). The resulting pipeline generated very complete assemblies, with 14 nuclear chromosomes represented by 28, 30, 20, and 21 nuclear contigs, respectively, for NF54, NF166.C8, 7G8, and NF135.C10, with each chromosome in the 3D7 reference represented by one to three contigs (Fig. 1). Several shorter contigs in NF54 (67,501 bps total), NF166.C8 (224,502 bps total), and NF135.C10 (80,944 bps total) could not be unambiguously assigned to an orthologous segment in the 3D7 reference genome; gene annotation showed that these contigs mostly contain members of multi-gene families and therefore are likely part of sub-telomeric regions. The cumulative lengths of the four assemblies ranged from 22.8 to 23.5 Mbp (Table 1), indicating variation in genome size among *P. falciparum* strains. In particular, the 7G8 assembly was several hundred thousand base-pairs smaller than the other three assemblies. To confirm that this was not an assembly error, we compared 7G8 to a previously published 7G8 PacBio-based assembly [32]. The two assemblies were extremely close in overall genome structure, differing only by ~ 25 kbp in cumulative length, and also shared a very similar number of SNP and small indel variants relative to 3D7 (Additional file 2: Table S4).

**Table 1:** The PfSPZ strains differ from the 3D7 in genome size and sequence. Characteristics of the Pacio assembly for each strain (first four columns), with the Pf 3D7 reference genome shown for comparison (italics). Single nucleotide polymorphisms (SNPs) and indels in each PfSPZ assembly as compared to 3D7, both genome-wide (All) or restricted to the core genome

Strain	# of nuclear contigs^1^	Cumulative length^2^	N50^3^	SNPs		Indels	
				All	Core^4^	All	Core^4^
*3D7*	*14*	*23,332,831*	*1,687,656*	*–*	*–*	*–*	*–*
NF54	28	23,404,633	1,527,116	1383	816	8396	7848
7G8	20	22,807,193	1,455,033	43,859	23,980	47,398	44,059
NF166.C8	30	23,281,895	1,610,926	51,011	25,925	47,259	43,335
NF135.C10	21	23,508,985	1,631,396	53,467	24,966	48,464	43,969

^1^Contigs: number of pieces of continuous sequence in the final assembly

^2^Cumulative length: total length of the contigs

^3^N50: length of the contig which, along with all contigs larger than itself, contain 50% of the assembly (larger numbers indicate a more complete assembly)

^4^Core genome as defined in [32]

### Structural variations in the genomes of the PfSPZ strains

Many structural variants (defined as indels or tandem repeat contractions or expansions, greater than 50 bp) were identified in each assembly by comparison to the 3D7 genome, impacting a cumulative length of 199.0 kbp in NF166.C8 to 340.9 kbp in NF135.C10 (Additional file 2: Table S5). Many smaller variants fell into coding regions (including known pre-erythrocytic antigens), often representing variation in repeat units (Additional file 3). Several larger structural variants (> 10 kbp) exist in 7G8, NF166.C8, and NF135.C10 relative to 3D7. Many of these regions contain members of multi-gene families, such as *var* genes (which encode PfEMP1 proteins), and as expected the number of *var* genes varied between each assembly (Additional file 4). While PfEMP1 proteins are most commonly studied in the context of blood-stage infections, several characteristics of these sequences may still be relevant for the interpretation of whole organism pre-erythrocytic vaccine trials. For example, NF166.C8 and NF135.C10 both had domain cassette sequences encoding DC8- and DC13-containing PfEMP1s, which have been associated with severe malaria [66], while 7G8 did not. In addition, a recently characterized PfEMP1 protein expressed on the surface of NF54 sporozoites (NF54*var*^sporo^) was shown to be involved in hepatocyte invasion (Pf3D7_0809100), and antibodies to this PfEMP1 blocked invasion [67]. No ortholog to NF54*var*^sporo^ was identified in the *var* repertoire of 7G8, NF166.C8, or NF135.C10; while there were *var* sequences in the three heterologous CHMI strains that contained the general domain structure (NTS-DBLa-CIDRa-DBLd-CIDRb) of NF54*var*^sporo^, none had its specific domain cassette (NTS-DBLα0.12-CIDRα2.2-DBLδ1-CIDRβ1) (Additional file 4). It remains to be determined whether a different, strain-specific, *var* gene fulfills a similar role in each of the heterologous PfSPZ strains.

Several other large structural variants impact regions housing non-multi-gene family members, although none is known to be involved in pre-erythrocytic immunity. Examples include a 31-kbp-long tandem expansion of a region of chromosome 12 in the 7G8 assembly (also present in the previously published assembly for 7G8 [32]) and a 22.7-kbp-long repeat expansion of a region of chromosome 5 in NF135.C10, both of which are supported by ~ 200 PacBio reads. The former is a segmental duplication containing a vacuolar iron transporter (PF3D7_1223700), a putative citrate/oxoglutarate carrier protein (PF3D7_1223800), a putative 50S ribosomal protein L24 (PF3D7_1223900), GTP cyclohydrolase I (PF3D7_1224000), and three conserved *Plasmodium* proteins of unknown function (PF3D7_1223500, PF3D7_1223600, PF3D7_1224100). The expanded region in NF135.C10 represents a tandem expansion of a segment housing the gene encoding the multidrug resistance protein PfMDR1 (PF3D7_0523000), resulting in a total of four copies of this gene in NF135.C10. Other genes in this tandem expansion include those encoding an iron-sulfur assembly protein (PF3D7_0522700), a putative pre-mRNA-splicing factor DUB31 (PF3D7_0522800), a putative zinc finger protein (PF3D7_0522900), and a putative mitochondrial-processing peptidase subunit alpha protein (PF3D7_0523100). In addition, the NF135.C10 assembly contained a large translocation involving chromosomes 7 (3D7 coordinates ~ 520,000 to ~ 960,000) and 8 (start to coordinate ~ 440,000) (Additional file 2: Figure S2). Since large synteny breaks are uncommon within and even between *Plasmodium* species, validation was done by generating Oxford Nanopore long-read data and building a Canu-based PacBio-Nanopore hybrid NF135.C10 assembly; in addition, several new PacBio-only assemblies were made, with different assembly programs (Additional file 2: Supplemental Text). All new assemblies supported a translocation event, although neither chromosome was resolved into a single supercontig. While an assembly artifact cannot be completely ruled out, the regions of chromosomes 7 and 8 where the translocation occurs are documented recombination hotspots that were identified specifically in isolates from Cambodia, the site of origin of NF135.C10 [68].

Several structural differences in genic regions were also identified between the NF54 assembly and the 3D7 genome (Additional file 3); if real, these structural variants would have important implications in the interpretation of trials using 3D7 as a homologous CHMI strain. For example, a 1887-bp tandem expansion was identified in the NF54 assembly on chromosome 10, which overlapped the region containing liver stage antigen 1 (PfLSA-1, PF3D7_1036400). The structure of this gene in the NF54 strain was reported when PfLSA-1 was first characterized, with unique N- and C-terminal regions flanking a repetitive region consisting of several dozen repeats of a 17 amino acid motif [69, 70]; the CDS of PfLSA-1 in the NF54 assembly was 5406 bp in length (matching the previously published sequence), but only 3489 bp long in the 3D7 reference. To determine if this was an assembly error in the NF54 assembly, the PfLSA-1 locus from a recently published PacBio-based assembly of 3D7 [31] was compared to that of NF54. The two sequences were identical, likely indicative of incorrect collapsing of the repeat region of PfLSA-1 in the 3D7 reference; NF54 and 3D7 PacBio-based assemblies had 79 units of the 17-mer amino acid repeat, compared to only 43 in the 3D7 reference sequence, a result further validated by the inconsistent depth of mapped Illumina reads from NF54 between the PfLSA repeat region and its flanking unique regions in the 3D7 reference (Additional file 2: Figure S3). Several other potential differences between NF54 and 3D7 were ruled out as remaining errors in the 3D7 assembly, several of which are present in a list of 3D7 reference patches recently published [33] (Additional file 3).

### Small sequence variants between PfSPZ strains and the reference 3D7 genome

Very few small sequence variants were identified in NF54 compared to the 3D7 reference; 17 non-synonymous mutations were present in 15 single-copy non-pseudogene-encoding loci (Additional file 5). Short indels were detected in 185 genes; many of these indels had a length that is not multiple of three and occurred in homopolymer runs, possibly representing remaining PacBio sequencing error. However, some may be real, as a small indel causing a frameshift in PF3D7_1417400, a putative protein-coding pseudogene that has previously been shown to accumulate premature stop codons in laboratory-adapted strains [71], and some may be of biologic importance, such as those seen in two histone-related proteins (PF3D7_0823300 and PF3D7_1020700). It has been reported that some clones of 3D7, unlike NF54, are unable to consistently produce gametocytes in long-term culture [26]; no SNPs were observed within or directly upstream of PfAP2-G (PF3D7_1222600) (Additional file 2: Table S6), which has been identified as a transcriptional regulator of sexual commitment in *P. falciparum* [72]. However, 7G8, NF66.C8, and NF135.C10 had numerous non-synonymous mutations and indels within putative AP2 genes (Additional file 2: Table S6). A non-synonymous mutation from arginine to proline (R1286P) was observed in an AP2-coincident C-terminal domain of PfAP2-L (PF3D7_0730300), a gene associated with liver stage development [73], in all PfSPZ strains compared to 3D7. Interestingly, NF135.C10 contained an insertion of almost 200 bp in length relative to 3D7 in the 3′ end of PfAP2-G; the insertion also carried a premature stop codon, leading to a considerably different C-terminal end for the transcription factor (Additional file 2: Figure S4). This alternate allele is also present in previously published assemblies for clones from Southeast Asia [32], including the culture-adapted strain Dd2, and variations of this insertion (without the in-frame stop codon) are also found in several non-human malaria *Plasmodium* species (Additional file 2: Figure S4), suggesting an interesting evolutionary trajectory of this sequence.

**Fig. 2:**
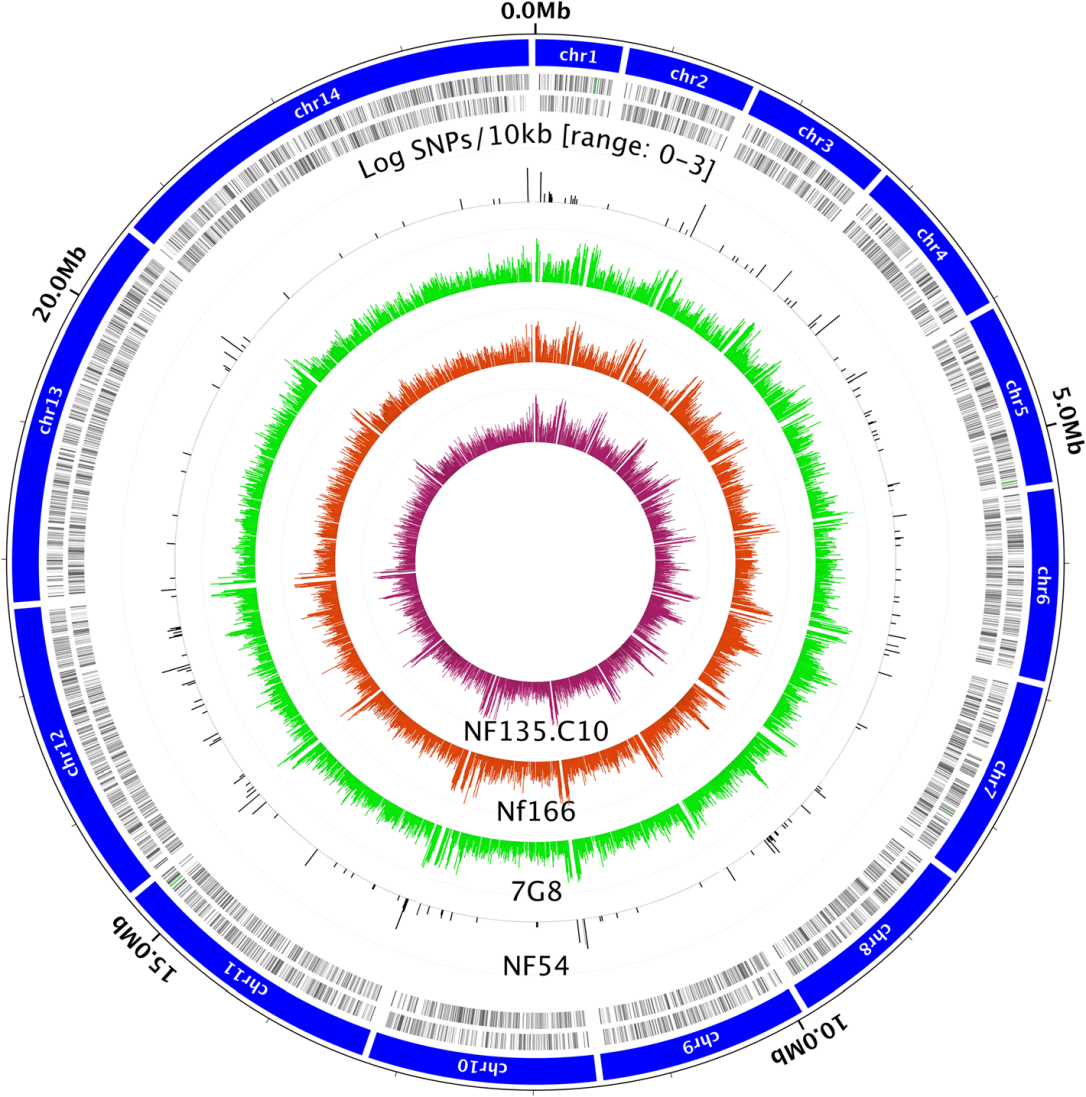
Distribution of polymorphisms in PfSPZ PacBio assemblies. Single nucleotide polymorphism (SNP) densities (log SNPs/ 10 kb) are shown for each assembly; the scale [0–3] refers to the range of the log-scaled SNP density graphs—from 10^0^ to 10^3^. Inner tracks, from outside to inside, are NF54 (black), 7G8 (green), NF166.C8 (orange), and NF135.C10 (pink). The outermost tracks are the 3D7 reference genome nuclear chromosomes (chrm1 to chrm 14, in blue), followed by 3D7 genes on the forward and reverse strand (black tick marks). Peaks in SNP densities mostly correlate with subtelomeric regions and internal multi-gene family clusters

Given that no absolute correlates of protection are known for whole organism *P. falciparum* vaccines, genetic differences were assessed both across the genome and in pre-erythrocytic genes of interest in the three heterologous CHMI strains. As expected, the number of mutations between 3D7 and these three PfSPZ strains was much higher than observed for NF54, with ~ 40–55 K SNPs and as many indels in each pairwise comparison. Indel length distributions showed distinct patterns in each strain (Additional file 2: Figure S5); the expected difference in the length distribution of small indels in coding versus non-coding regions across the genome suggests that most of the remaining indels correspond to true differences relative to 3D7. SNPs were roughly randomly distributed among intergenic regions, silent and non-synonymous sites (Table 1, Fig. 2), and corresponding to a pairwise SNP density relative to 3D7 of 1.9, 2.1 and 2.2 SNPs/kbp for 7G8, NF166.C8 and NF135.C10, respectively. Increased diversity was observed in regions known to house variable members of multi-gene families such as *var*s, *rifin*s, and *stevor*s. NF135.C10 had the highest number of unique SNPs genome-wide (SNPs not shared with other PfSPZ strains), with 5% more unique SNPs than NF166.C8 and 33% more than 7G8 (Additional file 2: Figure S6). A similar trend was seen when restricting the analyses to non-synonymous SNPs in the core genome (7.3% and 8% more than NF166.C8 and 7G8, respectively). The lower number of unique SNPs in 7G8 may be due in part to the smaller genome size of this strain.

SNPs were also common in a panel of 42 pre-erythrocytic genes known or suspected to be implicated in immunity to liver-stage parasites (see “Methods”; Additional file 2: Table S7). While the sequence of all these loci was identical between NF54 and 3D7, there was a wide range in the number of sequence variants per locus between 3D7 and the other three PfSPZ strains, with some genes being more conserved than others. For example, the circumsporozoite protein, PfCSP, showed 8, 7, and 6 non-synonymous mutations in 7G8, NF166.C8, and NF135.C10, respectively, relative to 3D7. However, PfLSA-1 had over 100 non-synonymous mutations in all three heterologous strains relative to 3D7 (many in the repetitive, difficult-to-align, region of this gene), in addition to significant length differences in the internal repeat region (Additional file 2: Figure S7).

### Immunological relevance of genetic variation among PfSPZ strains

**Fig. 3:**
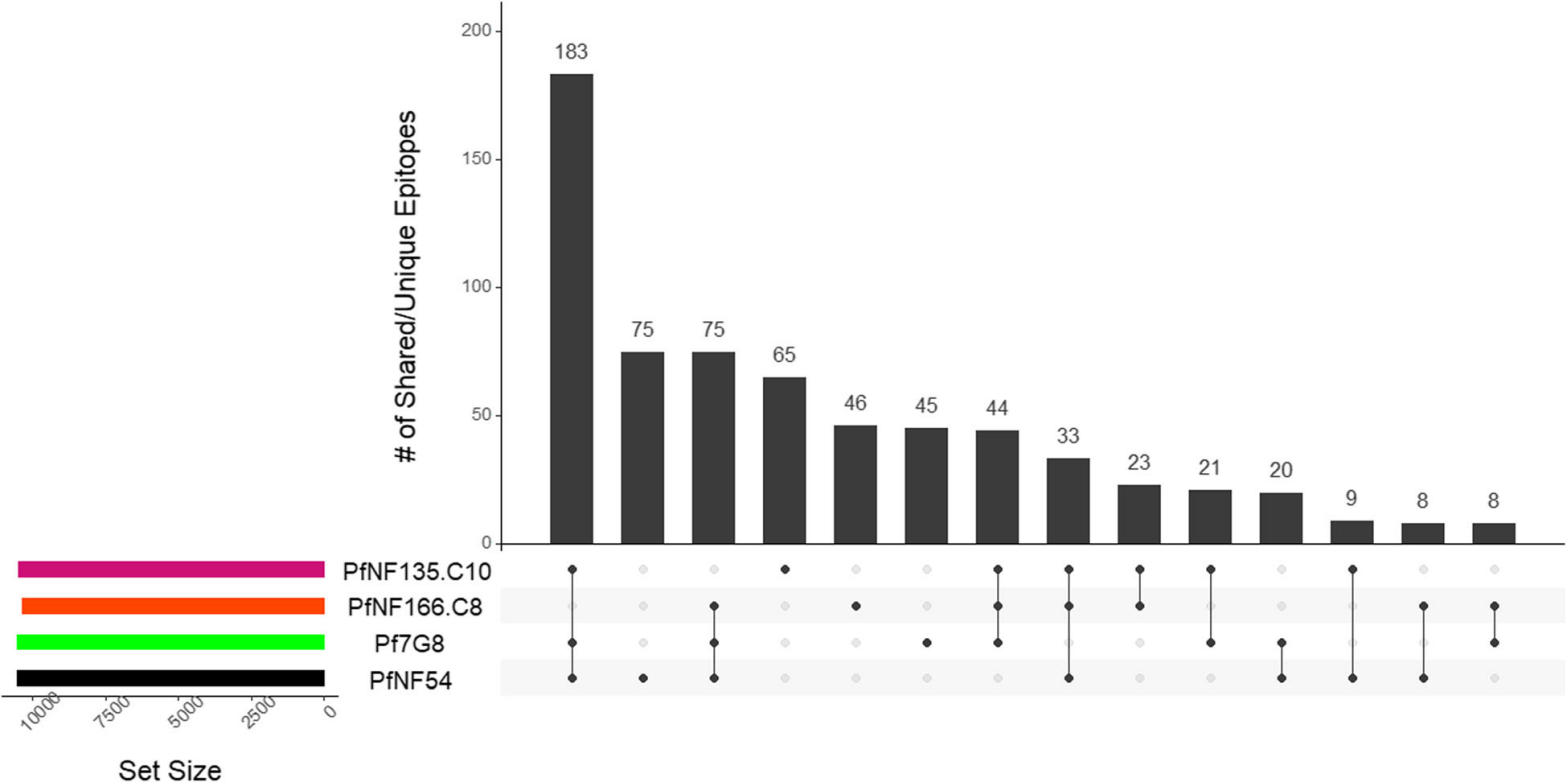
Comparison of predicted CD8^+^ T cell epitopes from pre-erythrocytic antigen amino acid sequences. CD8^+^ T cell epitopes were predicted in silico for 42 confirmed or suspected pre-erythrocytic antigens (See Additional file 2: Table S7 for a complete list of genes included in this analysis). The plot shows the number of shared or unique epitopes, as compared between different PfSPZ strain groupings. The height of the bar is the number of epitopes that fell into each intersection category, and the horizontal tracks below the bars show the PfSPZ strains that are included in that intersection. For example, the first bar represents the number of shared epitopes between NF54, 7G8, and NF135.C10. At the bottom left, colored tracks represent the total number of epitopes predicted across all genes (> 10 k for each strain). As the vast majority of predicted epitopes were shared among all four strains, that group was removed from the bar plot to achieve better visual definition for the other comparison

The sequence variants mentioned above may impact the ability of the immune system primed with NF54 to recognize the other PfSPZ strains, impairing vaccine efficacy against heterologous CHMI. Data from murine and non-human primate models [4, 27, 28, 74] demonstrate that CD8^+^ T cells are required for protective efficacy; therefore, the identification of shared and unique CD8^+^ T cell epitopes across the genome in all four PfSPZ strains may help interpret the differential efficacy seen in heterologous relative to homologous CHMI. We predicted CD8^+^ T cell epitopes in 42 genes whose product has been confirmed or suspected to be involved in pre-erythrocytic immunity (Fig. 3). Strong-binding MHC class I epitopes in the protein sequences from these loci were identified using in silico epitope predictions based on HLA types common in sub-Saharan Africa populations (Additional file 2: Table S1).

**Fig. 4:**
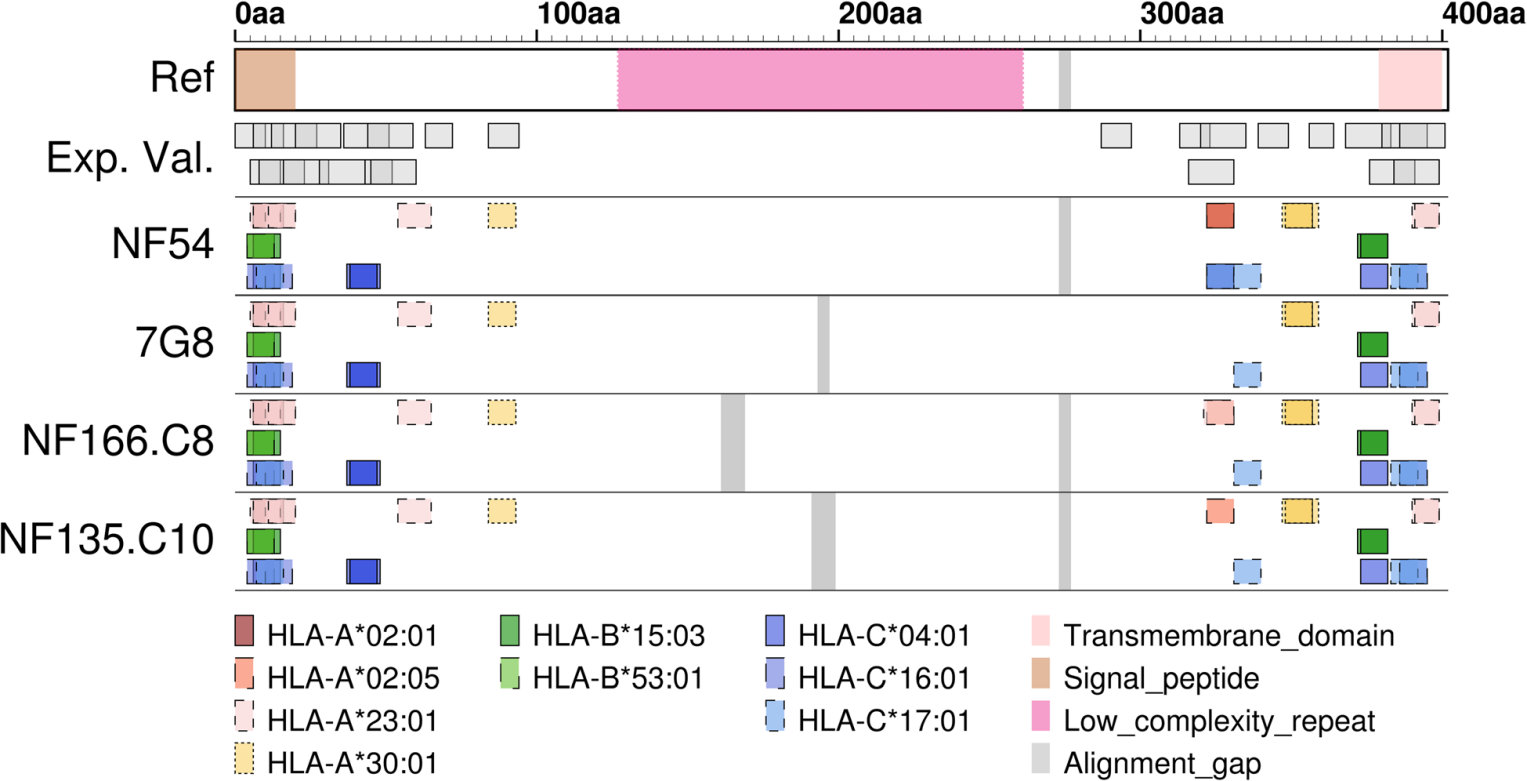
Predicted CD8^+^ T cell epitopes in the *P. falciparum* circumsporozoite protein (PfCSP). Protein domain information based on the 3D7 reference sequence of PfCSP is found in the first track. The second track are previously experimentally validated (Exp. Val.) epitopes (from [59], after removing duplicate epitope sequences and epitopes > 20 amino acids in length) and the following tracks are epitopes predicted in the PfCSP sequences of NF54, 7G8, NF166.C8, and NF135.C10, respectively. Each box is a sequence that was identified as an epitope, and colors represent the HLA type that identified the epitope. The experimentally validated epitopes do not have HLA types reflected and are simply jittered across two rows

Similar total numbers of epitopes (sum of unique epitopes, regardless of the HLA-type, across genes) were identified in the three heterologous CHMI strains, with each strain containing 10.5 K CD8^+^ T cell epitopes. NF54 had a slightly higher number of predicted epitopes compared to the other strains, possibly reflecting the slightly longer median sequence lengths in NF54 compared to the other strains (Additional file 2: Figure S8). While only a small number of CD8^+^ T cell epitopes, in a small number of antigens, have been experimentally validated [59], there was a strong overlap between these and the in silico-predicted epitopes. Only a small number of validated epitope sequences failed to overlap with the predicted epitope set (Fig. 4), at least one of which could be explained by differences in HLA types used in experiments and in silico predictions. The majority of predicted epitopes were shared across all four strains, reflecting epitopes predicted in conserved regions of the 42 genes used in this analysis. Of the three heterologous CHMI strains, NF135.C10 had the highest number of unique epitopes relative to all other strains (*n* = 65, Fig. 3) or to NF54 (*n* = 153, Additional file 2: Table S8). Both 7G8 and NF166.C8 had a similar number of unique epitopes (*n* = 45 and *n* = 46, respectively) and of epitopes not shared with NF54 (*n* = 117 and *n* = 121, respectively). Indels and repeat regions also sometimes affected the number of predicted epitopes in each antigen for each strain; for example, an insertion in 7G8 near amino acid residue 1600 in PfLISP-2 (PF3D7_0405300) contained additional predicted epitopes (Additional file 2: Figure S9). Similar patterns in variation in epitope recognition and frequency were found in other pre-erythrocytic genes of interest, including PfLSA-3 (PF3D7_0220000), PfAMA-1 (PF3D7_1133400), and PfTRAP (PF3D7_1335900) (Additional file 2: Figure S9).

Some of these variations in epitope sequences are relevant for the interpretation of the outcome of PfSPZ vaccine trials. For example, while all four strains are identical in sequence composition in a B cell epitope potentially relevant for protection recently identified PfCSP [75], another B cell epitope that partially overlaps it [76] contained an A98G amino acid difference in 7G8 and NF135.C10 relative to NF54 and NF166.C8. There was also variability in CD8^+^ T cell epitopes recognized in the Th2R region of the protein. Specifically, the PfCSP encoded by the 3D7/NF54 allele was predicted to bind to both HLA-A and HLA-C allele types, but the orthologous protein segments in NF166.C8 and NF135.C10 were only recognized by HLA-A allele types; notably, and given the HLA types studied, no epitope was detected at that position in PfCSP encoded in 7G8 (Fig. 4). Expansion of the analyses to additional HLA types revealed an allele (HLA-08:01) that is predicted to bind to the Th2R region of the 7G8-encoded PfCSP; however, HLA-08:01 is much more frequent in European populations (10–15%) than in African populations (1–6%) [56]. Therefore, if CD8^+^ T cell epitopes in the Th2R region of 7G8 are important for protection, which is currently unknown, the level of protection against CHMI with 7G8 observed in volunteers of European descent may not be informative of PfSPZ vaccine efficacy in Africa.

### PfSPZ strains and global parasite diversity

**Fig. 5:**
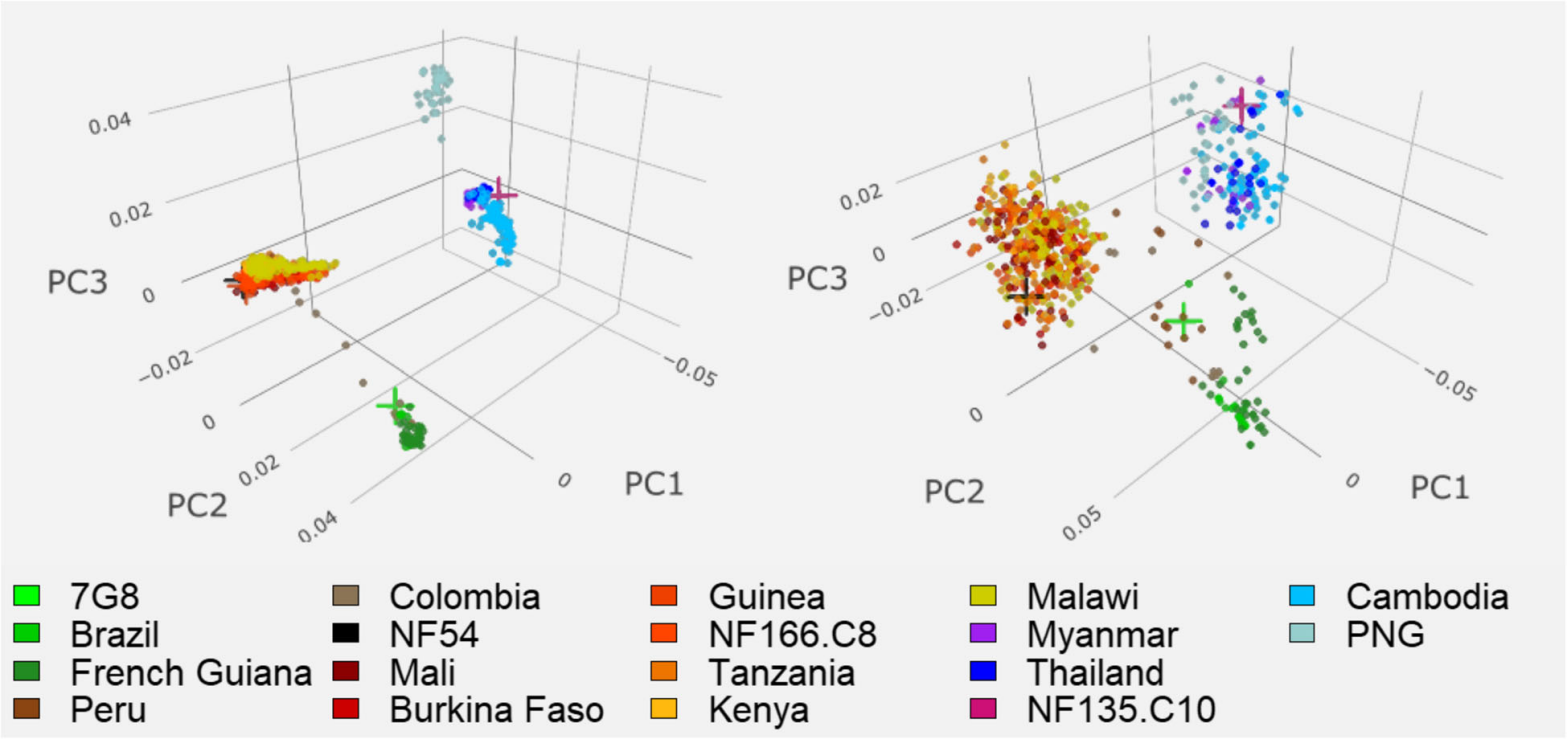
Global diversity of clinical isolates and PfSPZ strains. Principal coordinate analyses (PCoA) of clinical isolates (*n* = 654) from malaria-endemic regions and PfSPZ strains were conducted using biallelic non-synonymous SNPs across the entire genome (left, *n* = 31,761) and in a panel of 42 pre-erythrocytic genes of interest (right, *n* = 1060). For the genome-wide dataset, coordinate 1 separated South American and African isolates from Southeast Asian and Papua New Guinean isolates (27.6% of variation explained), coordinate two separated African isolates from South American isolates (10.7%), and coordinate three separated Southeast Asian isolates from Papua New Guinea (PNG) isolates (3.0%). Similar trends were found for the first two coordinates seen for the pre-erythrocytic gene data set (27.1 and 12.6%, respectively), but coordinate three separated isolates from all three regions (3.8%). In both datasets, NF54 (black cross) and NF166.C8 (orange cross) cluster with West African isolates (isolates labeled in red and dark orange colors), 7G8 (bright green cross) cluster with isolates from South America (greens and browns), and NF135.C10 (pink cross) clusters with isolates from Southeast Asia (purples and blues)

The four PfSPZ strains have been adapted and kept in culture for extended periods of time. To determine if they are still representative of the malaria-endemic regions from which they were collected, we compared these strains to over 600 recent (2007–2014) clinical isolates from South America, Africa, Southeast Asia, and Oceania (Additional file 1), using principal coordinates analysis (PCoA) based on SNP calls generated from Illumina whole genome sequencing data. The results confirmed the existence of global geographic differences in genetic variation previously reported [77, 78], including clustering by continent, as well as a separation of east from west Africa and of the Amazonian region from that west of the Andes (Fig. 5). The PfSPZ strains clustered with others from their respective geographic regions, both at the genome-wide level and when restricting the data set to SNPs in the panel of 42 pre-erythrocytic antigens, despite the long-term culturing of some of these strains (Fig. 5). An admixture analysis of South American and African clinical isolates confirmed that NF54 and NF166.C8 both have the genomic background characteristic of West Africa, while 7G8 is clearly a South American strain (Additional file 2: Figure S10).

NF135.C10 was isolated in the early 1990s [13], at a time when resistance to chloroquine and sulfadoxine-pyrimethamine resistance was entrenched and resistance to mefloquine was emerging [79, 80], and carries signals from this period of drug pressure. Four copies of PfMDR-1 were identified in NF135.C10 (Additional file 2: Table S9); however, two of these copies appeared to have premature stop codons introduced by SNPs and/or indels, leaving potentially only two functional copies in the genome. While NF135.C10 also had numerous point mutations relative to 3D7 in genes such as PfCRT (conveying chloroquine resistance), and PfDHPS and PfDHR (conveying sulfadoxine-pyrimethamine resistance), NF135.C10 was isolated before the widespread deployment of artemisinin-based combination therapies (ACTs) and had the wild-type allele in the locus that encodes the Kelch13 protein in chromosome 13 (PfK13) on chromosome 13, with no mutations known to convey artemisinin resistance detected in the propeller region (Additional file 2: Table S10).

**Fig. 6:**
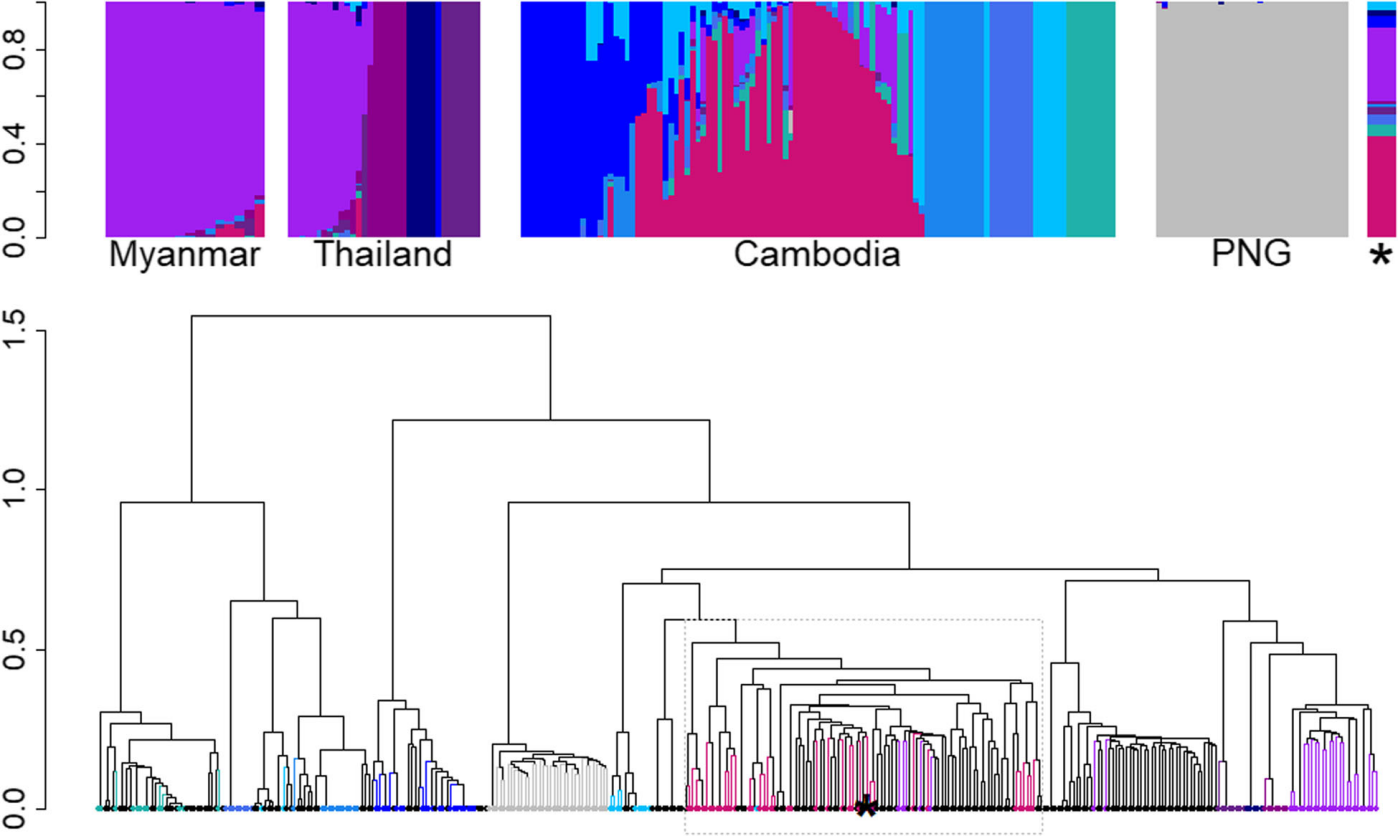
NF135.C10 is part of an admixed population of clinical isolates from Southeast Asia. Top: admixture plots for clinical isolates from Myanmar (*n* = 16), Thailand (*n* = 34), Cambodia (*n* = 109), Papua New Guinea (PNG, *n* = 34), and NF135.C10 (represented by a star) are shown. Each sample is a column, and the height of the different colors in each column corresponds to the proportion of the genome assigned to each *K* population by the model. Bottom: hierarchical clustering of the Southeast Asian isolates used in the admixture analysis (branch and leaves colored by their assigned subpopulation) and previously characterized Cambodian isolates (*n* = 167, black; [64]) place NF135.C10 (star) with samples from the previously identified KHA admixed population (shown in gray dashed box). The *y*-axis represents distance between clusters

The emergence in Southeast Asia of resistance to antimalarial drugs, including artemisinins and drugs used in artemisinin-based combination treatments (ACTs), is thought to underlie the complex and dynamic parasite population structure in the region [81]. Several relatively homogeneous subpopulations, whose origin is likely linked to the emergence and rapid spread of drug resistance mutations, exist in parallel with a sensitive subpopulation that reflects the ancestral population in the region (referred to as KH1), and another subpopulation of admixed genomic background (referred to as KHA), possibly the source of the drug-resistant subpopulations or the result of a secondary mix of resistant subpopulations [38, 64, 65, 82]. This has been accompanied by reports of individual K13 mutations conferring artemisinin resistance occurring independently on multiple genomic backgrounds [83]. To determine the subpopulation to which NF135.C10 belongs, an admixture analysis was conducted using isolates from Southeast Asia and Oceania, including NF135.C10. Eleven total populations were detected, of which seven contained Cambodian isolates (Fig. 6). Both admixture and hierarchical clustering analyses suggest that NF135.C10 is representative of the previously described admixed KHA subpopulation [64, 65] (Fig. 6), implying that NF135.C10 is representative of a long-standing admixed population of parasites in Cambodia rather than one of several subpopulations thought to have arisen recently in response to pressure from ACTs, an important observation if this strain is ever considered for use in a vaccination product.

## Discussion

Whole organism sporozoite vaccines have provided variable levels of protection in initial clinical trials; the radiation-attenuated PfSPZ vaccine has been shown to protect > 90% of subjects against homologous CHMI at 3 weeks after the last dose in 5 clinical trials in the USA [5, 7] and Germany [10]. However, efficacy has been lower against heterologous CHMI [7, 8], and in field studies in a region of intense transmission, in Mali, at 24 weeks [9]. Interestingly, for the exact same immunization regimen, protective efficacy by proportional analysis was greater in the field trial in Mali (29%) than it was against heterologous CHMI with Pf 7G8 in the USA at 24 weeks after last dose of vaccine (8%) [8, 10]. While evidence shows that whole organism-based vaccine efficacy can be improved by adjusting the vaccine dose and schedule [10], further optimization of such vaccines will be facilitated by a thorough understanding of the genotypic and immunologic differences among the PfSPZ strains and between them and parasites in malaria endemic regions.

A recent study examined whole genome short-read sequencing data to characterize NF166.C8 and NF135.C10 through SNP calls, and identified a number of non-synonymous mutations at a few loci potentially important for the efficacy of chemoprophylaxis with sporozoites, the foundation for PfSPZ-CVac [16]. The analyses described here, using high-quality de novo genome assemblies, expand the analysis to hard-to-call regions, such as those containing gene families, repeats, and other low complexity sequences. The added sensitivity enabled the thorough genomic characterization of these and additional vaccine-related strains, and revealed a considerably higher number of sequence variants than can be called using short read data alone, as well as indels and structural variants between assemblies. For example, the insertion close to the 3′ end of PfAP2-G detected in NF135.C10 and shared by Dd2 has not, to the best of our knowledge, been reported before, despite the multiple studies highlighting the importance of this gene in sexual commitment in *P. falciparum* strains, including Dd2 [72]. Long-read sequencing also confirmed that differences observed between the NF54 and 3D7 assemblies in a major liver stage antigen, PfLSA-1, represent one of a small number of errors lingering in the reference 3D7 genome, which is being continually updated and improved [33]. Confirmation that NF54 and 3D7 are identical at this locus is critical when 3D7 has been used as a homologous CHMI in whole sporozoite, NF54-based vaccine studies. Furthermore, the comprehensive sequence characterization of variant surface antigen-encoding loci, such as PfEMP1-encoding genes, will enable the use of the PfSPZ strains to study the role of these protein families in virulence, naturally acquired immunity and vaccine-induced protection [84].

The comprehensive genetic and genomic studies reported herein were designed to provide insight into the outcome of homologous and heterologous CHMI studies and to determine whether the CHMI strains can be used as a proxy for strains present in the field. Comparison of genome assemblies confirmed that NF54 and 3D7 have remained genetically very similar over time and that 3D7 is an appropriate homologous CHMI strain. As expected, 7G8, NF166.C8, and NF135.C10 were genetically very distinct from NF54 and 3D7, with thousands of differences across the genome including dozens in known pre-erythrocytic antigens. The identification of sequence variants (both SNPs and indels) within transcriptional regulators, such as the AP2 family, may assist in the study of different growth phenotypes in these strains. NF166.C8 and NF135.C10 merozoites enter the bloodstream several days earlier than those of NF54 [14], suggesting that NF54 may develop more slowly in hepatocytes than do the other two strains. Therefore, mutations in genes associated with liver-stage development (as was observed with PfAP2-L) may be of interest to explore further. Finally, comparison of the PfSPZ strains to whole genome sequencing data from clinical isolates shows that, at the whole genome level, they are indeed representative of their geographical regions of origin. We note, however, that potential transcriptional differences between PfSPZ and field strains, which could be caused by a small number of variants, remain to be explored.

These results can assist in the interpretation of CHMI studies in multiple ways. First, of the three heterologous strains, NF135.C10 is the most divergent from NF54, containing the highest numbers of unique SNPs and epitope sequences relative to the vaccine strain, which was expected from their respective geographic origins. However, results were less consistent for NF166.C8 and 7G8. Given its South American origin, 7G8 was expected to have more unique variants relative to NF54 than NF166.C8 did, but this was not always the case (for example, NF166.C8 had a slightly higher number of unique epitopes relative to NF54, compared to 7G8). These results show that the practice of equating geographic distance to genetic differentiation is not always valid and that the interpretation of CHMI studies should rest upon thorough genome-wide comparisons. Lastly, since, of all PfSPZ strains, NF135.C10 is the most genetically distinct from NF54, if proteome-wide genetic divergence is the primary determinant of differences in protection against different parasites, the extent to which NF54-based immunization protects against CHMI with NF135.C10 is important in understanding the ability of PfSPZ vaccine and other whole-organism malaria vaccines to protect against diverse parasites present world-wide. These conclusions are drawn from genome-wide analyses and from subsets of genes for which a role in whole-sporozoite-induced protection is suspected but not experimentally established. Conclusive statements regarding cross-protection will require the additional knowledge of the genetic basis of whole-organism vaccine protection.

Without more information on the epitope targets of protective immunity induced by PfSPZ vaccines, it is difficult to rationally design multi-strain PfSPZ vaccines. However, these data can potentially be used for the rational design of multi-strain sporozoite-based vaccines once knowledge of those critical epitope sequences is available. Characterization of a variety of *P. falciparum* strains may facilitate the development of region-specific or multi-strain vaccines with greater protective efficacy. Support for a genomics-guided approach to guide such next-generation vaccines can be found in other whole organism parasitic vaccines. Field trials testing the efficacy of first-generation whole killed-parasite vaccines against *Leishmania* had highly variable results [85]. While most studies failed to show protection, indicating that killed, whole-cell vaccines for leishmaniasis may not produce the necessary protective response, a trial demonstrating significant protection utilized a multi-strain vaccine, with strains collected from the immediate area of the trial [86], highlighting the importance of understanding the distribution of genetic diversity in pathogen populations. In addition, a highly efficacious non-attenuated, three-strain, whole organism vaccine exists against *Theileria parva*, a protozoan parasite that causes East coast fever in cattle. This vaccine, named Muguga Cocktail, consists of a mix of three live strains of *T. parva* that are administered in an infection-and-treatment method, similar to the approach utilized by PfSPZ-CVac. It has been shown recently that two of the strains are genetically very similar, possibly clones of the same isolates [87]. Despite this, the vaccine remains highly efficacious and in high demand [88]. In addition, the third vaccine strain in the Muguga Cocktail is quite distinct from the other two, with ~ 5 SNPs/kb [87], or about twice the SNP density seen between NF54 and other PfSPZ strains. These observations suggest that an efficacious multi-strain vaccine against a highly variable parasite species does not need to contain a large number of strains, but that the inclusion of highly divergent strains may be warranted. These results also speak to the promise of multi-strain vaccines against highly diverse pathogens, including apicomplexans with large genomes and complex life cycles.

## Conclusions

Next-generation whole genome sequencing technology has opened many avenues for infectious disease research and holds great promise for informing vaccine design. While most malaria vaccine development has occurred before the implementation of regular use of whole genome sequencing, the tools now available allow the precise characterization and informed selection of vaccine strains early in the development process. The results presented here will greatly assist these future research efforts, as well as aiding in the interpretation of clinical trials using the PfSPZ strains for vaccination and CHMI purposes.

## Supplementary information

Additional file 1. Samples used for analyses in the manuscript “Strains used in whole organism *Plasmodium falciparum* vaccine trials differ in genome structure, sequence, and immunogenic potential”. Excel spreadsheet listing all samples used in this analysis; if a sample was sequenced as part of a previously published study, the reference is listed (and are also included in the main text of the manuscript, see Methods and References sections), as well as accession IDs.

Additional file 2. Supplemental Text, Tables, and Figures. Word document containing supplemental information as cited in the main manuscript.

Additional file 3. Description of structural variants. Excel spreadsheet describing structural variants identified in each of the four PfSPZ strain assemblies.

Additional file 4. Description of *var* exon 1 sequences. Excel spreadsheet describing *var* exon 1 sequences recovered from each of the four PfSPZ strains.

Additional file 5. Description of variants found in the NF54 assembly. Excel spreadsheet listing SNP and indel differences found in the NF54 assembly and 3D7.

## Abbreviations

Bp: Base pair
CHMI: Controlled human malaria infection
HLA: Human leukocyte antigen
NCBI: National Center for Biotechnology Information
PacBio: Pacific Biosciences
PfSPZ: *P. falciparum* sporozoites
sWGA: Selective whole genome amplification
UPS: Upstream promoter

## Authors’ information

Additional author information for M. D. S, K. J., C. L., D. L. S (affiliated with the Armed Forces Research Institute of Medical Sciences): Material has been reviewed by the Walter Reed Army Institute of Research. There is no objection to its presentation and/or publication. The opinions or assertions contained herein are the private views of the author and are not to be construed as official or as reflecting true views of the Department of the Army or the Department of Defense. The investigators have adhered to the policies for protection of human subjects as prescribed in AR 70–25.
