# Supplementary material for: Strains used in whole organism Plasmodium falciparum vaccine trials differ in genome structure, sequence, and immunogenic potential
Source: Genome Med. 2020 Jan 8;12:6. doi: 10.1186/s13073-019-0708-9 (PMC6950926; doi:10.1186/s13073-019-0708-9)
Supplement: Supplementary file 2 — Supplemental Text, Tables, and Figures. Word document containing supplemental information as cited in the main manuscript. [file 13073_2019_708_MOESM2_ESM.docx]

**Title**: Strains used in whole organism *Plasmodium falciparum* vaccine trials differ in genome sequence, structure, and immunogenic potential

**Authors:** Kara A. Moser^1^, Elliott F. Drábek^1^, Ankit Dwivedi^1^, Emily M. Stucke^2^, Jonathan Crabtree^1^, Antoine Dara^2^, Zalak Shah^2^, Matthew Adams^2^, Tao Li^3^, Priscila T. Rodrigues^4^, Sergey Koren^5^, Adam M. Phillippy^5^, James B. Munro^1^, Amed Ouattara^2^, Benjamin C. Sparklin^1^, Julie C. Dunning Hotopp^1^, Kirsten E. Lyke^2^, Lisa Sadzewicz^1^, Luke Tallon^1^, Michele D. Spring^6^, Krisada Jongsakul^6^, Chanthap Lon^6^, David L. Saunders^6^, Marcelo U. Ferreira^4^, Myaing Nyunt^2§^, Miriam K. Laufer^2^, Mark Travassos^2^, Robert W. Sauerwein^7^, Shannon Takala-Harrison^2^, Claire M. Fraser^1^, B. Kim Lee Sim^3^, Stephen L. Hoffman^3^, Christopher V. Plowe^2§^, Joana C. Silva^1,8^

**Supplemental Text, Tables and Figures**

**Supplemental Text**

*Assembly polishing and validation of the assembly protocol*

The raw assembly of the NF54 genome, while containing 99.9% of the 3D7 genome in 30 contigs, showed ~78K sequence variants relative to 3D7 (**Figure S1**). Many of these differences were small (1-3) base pair (bp) indels (insertions or deletions), particularly in AT-rich regions and homopolymer runs (31% of indels involved only A/T base pairs and 51% of SNPs were A/T transversions), strongly indicative of remaining sequencing errors (1). To maximize error removal with existing tools, Quiver was run iteratively on the NF54 assembly, and the number of differences between NF54 and 3D7 was calculated at each step. Two consecutive Quiver runs consistently and substantially reduced the number of differences between NF54 and 3D7 (**Figure S1**). Additional Quiver iterations could further reduced the overall number of differences between NF54 and 3D7, but when Quiver was run similarly on the other three PfSPZ strains, no consistent pattern was observed past two Quiver iterations; therefore we used two Quiver iterations on all strains. This also had the added benefit of reducing the chance of over-fitting, i.e., “correcting” true differences between a strain and 3D7.

Illumina reads from each strain were used to further polish the assembly using Pilon. The resulting NF54 assembly was 23.4 Mb in length (**Figure 1**) and, when compared to the 3D7 reference, contains 8,396 indels and 1,383 single nucleotide polymorphisms (SNPs), a rate of ~59 SNPs per million base pairs (**Table 1**). In fact, only 17 non-synonymous mutations were detected in 15 intact protein-coding single-copy genes between the two genomes (**Supplemental Dataset 4**).

*Investigation of translocations through alternative sequencing and assembly* *techniques*

A large chromosomal rearrangement was observed in NF135.C10, where a middle section of chromosome 7 and end of chromosome 8 appeared to have changed places (**Figure S2**). However, the breakpoint areas were in repetitive regions containing multi-gene families, and were also not resolved in one piece (chromosome 7 and 8 were in three and two contigs, respectively, in the Canu assembly). In an attempt to validate this rearrangement, we generated additional long read, Oxford Nanopore data for NF135.C10, using the same DNA stock used previously for PacBio sequencing. The library was constructed with SQK-RAD004 and sequenced on a FLO-MIN106 R9 according to the manufacturer’s protocol except that sequencing beads were replaced with water. Bases were called with the ONT Albacore (version 2.2.4). Canu was then used as before (see Methods section) to build Nanopore-only and hybrid PacBio-Nanopore assemblies. Additionally, we generated two PacBio-only assemblies using hgap (2) and Falcon (3) (default parameters). The Nanopore run generated less data than our available PacBio dataset; we received 100,736 reads with an average read length of 3,121.5 bps from the single flowcell. The nanopore-only Canu assembly was therefore much more fragmented than our original Canu PacBio assembly (data not shown). However, the hybrid PacBio-Nanopore Canu assembly showed a modest improvement over the original assembly; while the regions in question were still not resolved in a continuous piece of sequence, chromosome 7 was contained in two (versus three) pieces (as did the PacBio-only Falcon assembly) (**Figure S2**). The new hybrid Canu assembly, as well as both PacBio-only Hgap and Falcon assemblies, still supported a rearrangement between a middle section of chromosome 7 and the end of chromosome 8 in NF135.C10 (**Figure S2**); however, no assembly managed to resolve either chromosome in a continuous piece of sequence, and each assembly had slight variations on how it resolved the rearrangement.

**Table S1:** Frequency of HLA types for major histocompatibility complex (MHC) class-1 epitopes in west and east Africa

|  | HLA Type - MHC Class 1^1^ | | | | | | | | |
| --- | --- | --- | --- | --- | --- | --- | --- | --- | --- |
|  | HLA-A02:01 | HLA-A02:05 | HLA-A23:01 | HLA-A30:01 | HLA-B15:03 | HLA-B53:01 | HLA-C04:01 | HLA-C16:01 | HLA-C17:01 |
| Mali | 0.083 |  | 0.228 | 0.141 | 0.069 | 0.159 | 0.213 | 0.283 | 0.143 |
| BF (Rimabaibe) | 0.138 |  |  |  | 0.138 |  |  | 0.16 | 0.106 |
| BF (Mossi) |  |  |  |  |  | 0.208 |  | 0.179 | 0.151 |
| BF (Fulani) |  | 0.092 |  |  |  | 0.082 |  | 0.214 | 0.02 |
| Equatorial Guinea | |  |  |  |  |  |  | 0.105 |  |
| Tanzania |  |  |  |  | 0.143 | 0.091 |  |  |  |
| Kenya (Luo) | 0.115 | 0.087 | 0.089 | 0.084 | 0.089 | 0.068 | 0.132 | 0.045 | 0.087 |
| Kenya (Nandi) | 0.118 |  |  |  | 0.079 | 0.088 | 0.115 | 0.044 | 0.102 |
| ^1^Allele frequencies queried from the Allele Frequency database (main manuscript reference 56, accessed October 2016 and from the literature (main manuscript references 57 and 58). BF: Burkina Faso. | | | | | | | |  |  |

**Table S2:** Pacific Biosciences (PacBio) long-read (P6-P4 Chemistry) whole genome sequencing data

| Strain | Average Library Size  (bp) | # of SMRT Cells | Total # of Reads | Total # of bp | Mean Read Length  (bp) | Max Read Length  (bp) | Coverage^1^ |
| --- | --- | --- | --- | --- | --- | --- | --- |
| NF54 | 18,800 | 4 | 557,996 | 4,146,771,099 | 7,432 | 51,499 | 151.6 |
| NF166.C8 | 19,749 | 4 | 768,860 | 5,267,844,194 | 6,856 | 49,121 | 194.5 |
| 7G8 | 19,490 | 4 | 292,390 | 2,558,840,554 | 8,754 | 55,366 | 99.1 |
| NF135.C10 | 17,035 | 4 | 357,521 | 2,768,837,384 | 7,757 | 50,024 | 106.9 |
| ^1^Calculated by the average read length multiplied by number of reads that mapped to the 3D7 reference (PlasmoDBv24), divided by 3D7 genome size | | | | | | | |

**Table S3:** Illumina short-read whole genome sequencing data (HiSeq 2500/4000)

| Strain | Average Library Size  (bp) | Read Length  (bp) | Total # of Reads | Total # of bp | Coverage^1^ |
| --- | --- | --- | --- | --- | --- |
| NF54 | 403 | 100 | 39,384,560 | 3,977,840,560 | 169 |
| NF166.C8 | 359 | 150 | 58,890,306 | 8,892,436,206 | 263 |
| 7G8 | 322 | 150 | 54,894,060 | 8,289,003,060 | 340 |
| NF135.C10 | 330 | 150 | 56,189,696 | 8,484,644,096 | 268 |
| ^1^Calculated by the average read length multiplied by number of reads that mapped to the 3D7 reference (PlasmoDBv24), divided by 3D7 genome size | | | | | |

**Table S4:** Comparison of 7G8 assembly generated for this manuscript (mns.) to a previously generated 7G8 assembly available on PlasmoDB (v41) (reference 32 in main manuscript). Variants were identified by comparing each assembly to the 3D7 reference genome using nucmer’s show-snps function (see Methods). Nm3=non-multiple of 3; m3=multiple of 3.

| Genomic Partition | Type of Variant | Mns. | PlasmoDB |
| --- | --- | --- | --- |
| Coding | deletion.Nm3 | 1,088 | 1,171 |
|  | deletion.m3 | 2,391 | 2,369 |
|  | insertion.Nm3 | 1,125 | 1,040 |
|  | insertion.m3 | 2,143 | 2,150 |
|  | point mutation | 21,452 | 21,165 |
| Non-coding | deletion.Nm3 | 16,094 | 17,411 |
|  | deletion.m3 | 3,616 | 3,703 |
|  | insertion.Nm3 | 17,160 | 16,009 |
|  | insertion.m3 | 3,616 | 3,545 |
|  | point mutation | 22,907 | 22,694 |

**Table S5:** Structural variants (>50 bps) in each PfSPZ assembly as compared to the 3D7 reference genome^1^, including number and cumulative length in base pairs.

| Variant Type | NF54 | | 7G8 | | NF166.C8 | | NF135.C10 | |
| --- | --- | --- | --- | --- | --- | --- | --- | --- |
|  | number | bp | number | bp | number | bp | number | bp |
| Insertions | 4 | 476 | 170 | 34,060 | 161 | 16,782 | 180 | 31,721 |
| Deletions | 2 | 131 | 187 | 16,030 | 156 | 22,860 | 209 | 21,654 |
| Tandem_expansion | 26 | 23,672 | 102 | 61,958 | 114 | 95,085 | 118 | 162,337 |
| Tandem_contraction | 5 | 1,416 | 137 | 22,077 | 110 | 31,885 | 135 | 20,872 |
| Repeat_expansion | 2 | 738 | 19 | 12,500 | 22 | 14,070 | 23 | 20,526 |
| Repeat_contraction | 0 | 0 | 21 | 54,963 | 15 | 18,364 | 27 | 83,759 |
| **Total** | **39** | **26,433** | **636** | **201,318** | **578** | **199,046** | **692** | **340,869** |

^1^A small number of structural variants detected here may be due to existing errors in the 3D7 reference genome (see Additional File 3).

**Table S6:** Variants identified in loci encoding predicted AP2 transcription factors

| Gene ID | Product Description | NF54 | | | 7G8 | | | NF166.C8 | | | NF135.C10 | | | Mutations occur in AP2 domains? |
| --- | --- | --- | --- | --- | --- | --- | --- | --- | --- | --- | --- | --- | --- | --- |
|  |  | SNPs | | AA Length Difference | SNPs | | AA Length Difference | SNPs | | AA Length Difference | SNPs | | AA Length Difference |  |
|  |  | S | NS |  | S | NS |  | S | NS |  | S | NS |  |  |
| PF3D7_0404100 | AP2 domain transcription factor AP2-SP2, putative | 0 | 0 | 0 | 8 | 6 | -14 | 10 | 5 | -21 | 1 | 6 | -52 | no |
| PF3D7_0420300 | AP2 domain transcription factor, putative | 0 | 0 | 0 | 4 | 5 | 15 | 5 | 7 | 18 | 4 | 7 | -21 | 7G8[3274:H->Y] |
| PF3D7_0516800 | AP2 domain transcription factor AP2-O2, putative | 0 | 0 | 0 | 2 | 6 | 52 | 2.5 | 7.5 | 80 | 2.5 | 9.5 | 62 | no |
| PF3D7_0604100 | AP2 domain transcription factor | 0 | 0 | 0 | 0 | 1 | 13 | 0 | 1 | -3 | 0 | 1 | -2 | no |
| PF3D7_0611200 | AP2 domain transcription factor, putative | 0 | 0 | 0 | 1 | 0 | 0 | 0 | 0 | 0 | 0 | 0 | 0 | no |
| PF3D7_0613800 | AP2 domain transcription factor, putative | 0 | 0 | -1 | 9.5 | 28.5 | -9 | 12.5 | 14.5 | -5 | 9.5 | 25.5 | 24 | 7G8, NF166, NF135.C10[3713:N->D] |
| PF3D7_0622900 | AP2 domain transcription factor AP2Tel | 0 | 0 | 0 | 0 | 0 | 10 | 0 | 0 | -6 | 0 | 0 | -2 | no |
| PF3D7_0730300 | AP2 domain transcription factor AP2-L | 0 | 1 | 0 | 1 | 3 | 8 | 1 | 3 | 2 | 2 | 4 | 13 | no |
| PF3D7_0802100 | AP2 domain transcription factor, putative | 0 | 0 | 0 | 5 | 6 | 10 | 2 | 3 | -6 | 2 | 2 | -8 | no |
| PF3D7_0934400 | AP2 domain transcription factor, putative | 0 | 0 | 0 | 0 | 0 | 0 | 0 | 1 | 0 | 0 | 0 | 0 | no |
| PF3D7_1007700 | AP2 domain transcription factor, putative, AP2-I | 0 | 0 | 0 | 0 | 0 | 19 | 0 | 1 | 27 | 1.5 | 4.5 | 37 | no |
| PF3D7_1107800 | AP2 domain transcription factor, putative | 0 | 0 | 0 | 0 | 1 | -5 | 1 | 0 | -7 | 0 | 2 | 1 | no |
| PF3D7_1115500 | AP2 domain transcription factor, putative | 0 | 0 | 0 | 0 | 0 | 0 | 0 | 0 | 0 | 0 | 0 | 0 | no |
| PF3D7_1139300 | AP2 domain transcription factor, putative | 0 | 0 | 0 | 1 | 7 | -9 | 1 | 5 | -4 | 1 | 6 | -5 | no |
| PF3D7_1143100 | AP2 domain transcription factor AP2-O, putative | 0 | 0 | 0 | 0 | 3 | -1 | 0 | 2 | -21 | 0 | 3 | -29 | no |
| PF3D7_1222400 | AP2 domain transcription factor | 0 | 0 | 0 | 3 | 2 | 11 | 3 | 2 | 9 | 1 | 3 | -13 | no |
| PF3D7_1222600 | AP2 domain transcription factor AP2-G | 0 | 0 | 0 | 1 | 7 | 1 | 1 | 7 | 2 | 1 | 7 | 70 | no |
| PF3D7_1239200 | AP2 domain transcription factor, putative | 0 | 0 | 0 | 0 | 0 | 5 | 0 | 1 | 1 | 0 | 1 | 1 | no |
| PF3D7_1305200 | AP2 domain transcription factor, putative | 0 | 0 | 0 | 0 | 0 | 0 | 0 | 0 | 0 | 0 | 0 | 0 | no |
| PF3D7_1317200 | AP2 domain transcription factor, putative | 0 | 0 | -1 | 0 | 0 | -22 | 0 | 0 | -37 | 0 | 0 | -38 | no |
| PF3D7_1342900 | AP2 domain transcription factor, putative | 0 | 0 | 0 | 1 | 4 | 12 | 3 | 9 | -19 | 1 | 5 | -69 | no |
| PF3D7_1350900 | AP2 domain transcription factor AP2-O4, putative | 0 | 0 | 0 | 1 | 8 | -4 | 2 | 8 | -5 | 3 | 8 | -5 | - |
| PF3D7_1408200 | AP2 domain transcription factor AP2-G2, putative | 0 | 0 | 0 | 1 | 8 | -8 | 2 | 8 | -16 | 3 | 8 | -39 | no |
| PF3D7_1429200 | AP2 domain transcription factor AP2-O3, putative | 0 | 0 | 0 | 0 | 0 | 18 | 0 | 0 | 6 | 0 | 0 | -6 | no |
| PF3D7_1449500 | AP2 domain transcription factor, putative | 0 | 0 | 0 | 2 | 1 | 2 | 1 | 1 | -1 | 1 | 1 | 4 | no |
| PF3D7_1456000 | AP2 domain transcription factor, putative | 0 | 0 | 0 | 0 | 2 | -11 | 1 | 4 | 16 | 3 | 2 | -16 | no |
| PF3D7_1466400 | AP2 domain transcription factor AP2-SP | 0 | 0 | 0 | 4 | 5 | 0 | 5 | 7 | 1 | 4 | 7 | 0 | no |

**Table S7:** Variants identified in potentially important pre-erythrocytic loci in 7G8, NF166.C8, and NF135.C10

| Gene ID | Product Description | Gene Panel^1^ | | | 7G8 | | NF166.C8 | | NF135.C10 | |
| --- | --- | --- | --- | --- | --- | --- | --- | --- | --- | --- |
|  |  | Pre-erythroctyic Antigens | PfSPZ Vaccinees | PfSPZ-CVac Vaccinees | Synonymous (S) and Non-synonymous (NS) Variants | | | | | |
|  |  |  |  |  | S | NS | S | NS | S | NS |
| PF3D7_0206900.2 | merozoite surface protein 5 (MSP5) | x | x |  | 0 | 1 | 1 | 0 | 0 | 0 |
| PF3D7_0207000 | merozoite surface protein 4 (MSP4) | x |  | x | 0 | 3 | 0 | 0 | 0 | 0 |
| PF3D7_0220000 | liver stage antigen 3 (LSA3) | x |  |  | 12 | 42 | 13 | 29 | 12 | 62 |
| PF3D7_0304600 | circumsporozoite protein (CSP) | x | x | x | 33 | 8 | 4 | 7 | 2 | 6 |
| PF3D7_0312400 | pfGSK3 |  | x |  | 0 | 0 | 0 | 0 | 0 | 1 |
| PF3D7_0405300 | liver specific protein 2 (LISP2) | x |  | x | 10 | 33 | 11 | 29 | 3 | 21 |
| PF3D7_0408600 | sporozoite invasion-associated protein 1 (SIAP1) | x |  |  | 0 | 4 | 0 | 3 | 0 | 5 |
| PF3D7_0408700 | perforin-like protein 1 (PLP1/SPECT2) | x |  |  | 1 | 1 | 1 | 2 | 0 | 2 |
| PF3D7_0420000 | Zinc finger protein, putative |  |  | x | 7 | 19 | 7 | 11 | 7 | 16 |
| PF3D7_0522400 | Conserved Plasmodium protein, unknown function |  |  | x | 0 | 5 | 3 | 8 | 5 | 10 |
| PF3D7_0725100 | Conserved Plasmodium membrane protein, unknown function |  |  | x | 1 | 1 | 1 | 0 | 1 | 0 |
| PF3D7_0726400 | Conserved Plasmodium membrane protein, unknown function |  |  | x | 2 | 9 | 4 | 7 | 2 | 8 |
| PF3D7_0728600 | zinc finger, C3HC4 type, putative |  | x |  | 1 | 2 | 4 | 2 | 3 | 5 |
| PF3D7_0812300 | sporozoite surface protein 3 (SSP3) | x |  |  | 0 | 0 | 0 | 1 | 0 | 1 |
| PF3D7_0815500 | Conserved Plasmodium protein, unknown function |  |  | x | 0 | 0 | 0 | 0 | 0 | 0 |
| PF3D7_0826100 | E3 ubiquitin-protein ligase, putative |  |  | x | 4 | 7 | 3 | 8 | 10 | 15 |
| PF3D7_0828100 | conserved Plasmodium protein, unkown function |  | x |  | 1 | 2 | 1 | 1 | 2 | 2 |
| PF3D7_0830300 | sporozoite invasion-associated protein-2 (SIAP-2) | x |  |  | 0 | 4 | 0 | 4 | 0 | 5 |
| PF3D7_0906700 | Leucine-rich repeat protein (PfLR9) |  | x |  | 0 | 0 | 0 | 0 | 0 | 0 |
| PF3D7_1021700 | Conserved Plasmodium membrane protein, unknown function |  |  | x | 3 | 12 | 3 | 6 | 4 | 10 |
| PF3D7_1030200 | claudin-like apicomplexan microneme protein, putative |  | x |  | 2 | 1 | 1 | 0 | 1 | 0 |
| PF3D7_1035300 | Glutamate-rich protein (GLURP) |  |  | x | 4 | 18 | 1 | 2 | 6 | 28 |
| PF3D7_1036400* | Liver stage antigen 1 (LSA1) | x |  | x | > 100 | > 100 | > 100 | 89 | > 100 | > 100 |
| PF3D7_1121600 | exported protein 1 (EXP1) | x |  |  | 0 | 1 | 0 | 1 | 0 | 2 |
| PF3D7_1133400 | apical membrane antigen 1 (AMA1) | x |  |  | 1 | 31 | 3 | 30 | 3 | 31 |
| PF3D7_1138400 | Guanylyl cyclase (GCalpha) |  |  | x | 1 | 3 | 1 | 5 | 2 | 7 |
| PF3D7_1147000 | sporozoite asparagine-rich protein (SLARP) | x |  |  | 2 | 3 | 1 | 3 | 4 | 6 |
| PF3D7_1216600 | cell traversal protein for ookinetes and sporozoites (CelTOS) | x |  |  | 0 | 10 | 0 | 4 | 0 | 7 |
| PF3D7_1229100 | Multidrug resistance-associated protein 2 (MRP2) |  |  | x | 2 | 6 | 1 | 4 | 2 | 7 |
| PF3D7_1243900 | double c2-like domain-containing protein (PfD0C2) |  | x |  | 8 | 10 | 3 | 6 | 2 | 8 |
| PF3D7_1318300 | Conserved Plasmodium protein, unknown function |  |  | x | 2 | 4 | 3 | 5 | 0 | 3 |
| PF3D7_1325900 | Conserved Plasmodium protein, unknown function |  |  | x | 7 | 12 | 2 | 7 | 1 | 9 |
| PF3D7_1335900 | thrombospondin-related anonymous protein (TRAP) | x |  |  | 0 | 21 | 0 | 20 | 0 | 16 |
| PF3D7_1342500 | sporozoite protein essential for cell traversal (SPECT1) | x |  |  | 0 | 2 | 0 | 0 | 0 | 1 |
| PF3D7_1349300 | Tyrosine kinase-like protein (TKL3) |  |  | x | 2 | 10 | 1 | 5 | 1 | 12 |
| PF3D7_1365300 | Conserved Plasmodium protein, unknown function |  |  | x | 0 | 0 | 1 | 2 | 0 | 4 |
| PF3D7_1405400 | DNA mismatch repair protein, putative |  |  | x | 0 | 0 | 0 | 0 | 0 | 0 |
| PF3D7_1408700 | Conserved Plasmodium protein, unknown function |  |  | x | 2 | 7 | 2 | 8 | 4 | 6 |
| PF3D7_1438800 | conserved Plasmodium protein, unknown function |  | x |  | 0 | 0 | 0 | 0 | 0 | 0 |
| PF3D7_1465800 | Dynein beta chain, putative |  |  | x | 3 | 4 | 4 | 6 | 4 | 7 |
| PF3D7_1468100 | conserved Plasmodium protein, unknown function |  | x |  | 2 | 2 | 2 | 0 | 2 | 4 |
| PF3D7_1469600 | Biotin carboxylase subunit of acetyl CoA carboxylase, putative (ACC) |  |  | x | 4 | 8 | 0 | 12 | 1 | 12 |
| ^1^Three sets of genes were chosen for variant identification. The first was a list of 16 genes identified in the literature as potential pre-erythrocytic antigens (Pre-erythrocytic antigens). The other two categories were genes by sera from PfSPZ Vaccine and PfSPZ-CVac vaccinees (PfSPZ Vaccinees, PfSPZ-CVac Vaccinees). | | | | | | | | | | |
| ** The large number of SNPs in LSA-1 is possibly a reflection of the difficulty aligning the variable repeat region units in this gene. | | | | | | | | | | |

**Table S8:** Number of Unique Epitopes When Compared to NF54

| Gene ID | Product Description | Number of Unique Epitopes | | |
| --- | --- | --- | --- | --- |
|  |  | 7G8 | NF166.C8 | NF135.C10 |
| PF3D7_0220000 | liver stage antigen 3 (LSA3) |  | 3 | 4 |
| PF3D7_0304600 | circumsporozoite protein (CSP) |  | 2 | 1 |
| PF3D7_0312400 | pfGSK3 |  |  | 2 |
| PF3D7_0405300 | liver specific protein 2 (LISP2, sequestrin) | 9 | 20 | 18 |
| PF3D7_0408600 | sporozoite invasion-associated protein 1 (SIAP1) | 5 | 5 | 11 |
| PF3D7_0420000 | zinc finger protein, putative | 9 | 5 | 7 |
| PF3D7_0522400 | conserved Plasmodium protein, unknown function | 1 | 7 | 4 |
| PF3D7_0725100 | conserved Plasmodium membrane protein, unknown function | 6 |  |  |
| PF3D7_0726400 | conserved Plasmodium membrane protein, unknown function | 8 | 5 | 6 |
| PF3D7_0728600 | zinc finger, C3HC4 type, putative | 2 |  | 2 |
| PF3D7_0826100 | E3 ubiquitin-protein ligase, putative | 2 |  | 4 |
| PF3D7_0828100 | conserved Plasmodium protein, unkown function | 3 | 3 | 7 |
| PF3D7_0830300 | sporozoite invasion-associated protein-2 (SIAP-2) | 5 | 4 | 5 |
| PF3D7_1021700 | conserved Plasmodium membrane protein, unknown function | 1 | 2 | 1 |
| PF3D7_1030200 | claudin-like apicomplexan microneme protein, putative | 2 | 2 | 2 |
| PF3D7_1035300 | glutamate-rich protein (GLURP) | 2 |  | 1 |
| PF3D7_1036400 | liver stage antigen 1 (LSA1) | 3 | 3 | 1 |
| PF3D7_1133400 | apical membrane antigen 1 (AMA1) | 16 | 12 | 15 |
| PF3D7_1138400 | guanylyl cyclase (GCalpha) | 1 | 1 | 1 |
| PF3D7_1147000 | sporozoite asparagine-rich protein (SLARP) | 1 | 3 | 1 |
| PF3D7_1216600 | cell traversal protein for ookinetes and sporozoites (CelTOS) |  | 3 | 1 |
| PF3D7_1229100 | multidrug resistance-associated protein 2 (MRP2) | 9 | 5 | 25 |
| PF3D7_1243900 | double c2-like domain-containing protein (PfD0C2) | 1 |  | 3 |
| PF3D7_1318300 | conserved Plasmodium protein, unknown function | 2 | 2 |  |
| PF3D7_1335900 | thrombospondin-related anonymous protein (TRAP) | 4 | 4 | 3 |
| PF3D7_1342500 | sporozoite protein essential for cell traversal (SPECT1) | 5 |  | 2 |
| PF3D7_1365300 | conserved Plasmodium protein, unknown function |  |  | 7 |
| PF3D7_1408700 | conserved Plasmodium protein, unknown function | 12 | 14 | 12 |
| PF3D7_1465800 | dynein beta chain, putative | 3 | 7 | 4 |
| PF3D7_1469600 | biotin carboxylase subunit of acetyl CoA carboxylase, putative (ACC) | 5 | 8 | 3 |
| *Number of Unique Epitopes* | | 117 | 121 | 153 |

**Table S9**: Copy number of known drug resistance genes in the four PfSPZ strains

| Gene ID | Product | Drug | NF54 | 7G8 | NF166.C8 | NF135.C10 |
| --- | --- | --- | --- | --- | --- | --- |
|  |  |  |  |  |  |  |
| PF3D7_0417200 | PfDHFR | sulfadoxine-pyrimethamine | 1 | 1 | 1 | 1 |
| PF3D7_0523000 | PfMDR1 | artesunate, piperaquine, quinine, artemisinin | 1 | 1 | 1 | 4 |
| PF3D7_0709000 | PfCRT | chloroquine | 1 | 1 | 1 | 1 |
| PF3D7_0810800 | PfDHPS | sulfadoxine-pyrimethamine |  |  |  |  |
| PF3D7_1224000 | PfGHC1 | sulfadoxine-pyrimethamine | 4 | 2 | 1 | 3 |
| PF3D7_1343700 | PfKELCH13 | artemisinin |  |  |  |  |
| PF3D7_1408000 | PfPLASMEPSIN-2 | piperaquine | 1 | 1 | 1 | 1 |
| PF3D7_1408100 | PfPLASMEPSIN-3 | piperaquine | 1 | 1 | 1 | 1 |
| mal_mito_3 | PfCTYB | atovaquone | 1 | 1 | 1 | 1 |

**Table S10:** Non-synonymous SNPs in known drug resistance genes in the four PfSPZ strains (bolded indicates a codon change at that position has previously been shown to be associated with resistance)

| Gene ID | Product | Drug | NF54 | 7G8 | NF166.C8 | NF135.C10 |
| --- | --- | --- | --- | --- | --- | --- |
|  |  |  |  |  |  |  |
| PF3D7_0417200 | PfDHFR | sulfadoxine-pyrimethamine | - | **N51I**, **S108N** | **N51I**, **C59R**, **S108N** | **N51I**, **C59R**, **S108N**, **I164L** |
| PF3D7_0523000 | PfMDR1 | chloroquine, quinine, mefloquine, amodiaquine, artemisinin | - | **Y184F, S1034C, N1042D, D1246Y** | **Y184F** | F1226Y |
| PF3D7_0709000 | PfCRT | chloroquine | - | **C72S, K76T**, **A220S, N326D, I356L** | - | **M74I, N75E,** **K76T**, **A220S, Q271E, N326S,** **I356T**, **R371I** |
| PF3D7_0810800 | PfDHPS | sulfadoxine-pyrimethamine | - | - | **G437A*** | **S436A**, **K540E** |
| PF3D7_1343700 | PfKELCH13 | artemisinin | - | - | K189T | - |
| mal_mito_3 | PfCYTB | atovaquone | - | - | - | - |

*Reference allele in this case conveys resistance; therefore, NF166.C8 carries the allele conveying sensitivity, while the other strains match 3D7 in this position (and therefore carry the allele conveying resistance).


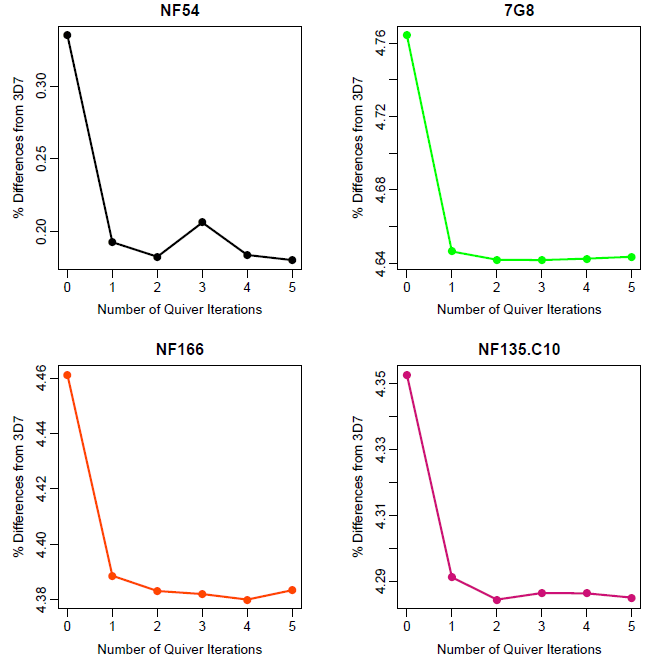


**Fig. S1: Polishing PfSPZ assemblies iteratively with Quiver minimized errors in the assemblies.** PacBio and Illumina reads were used to polish the PfSPZ assemblies with Quiver and Pilon, respectively. To minimize the remaining sequencing errors in the assemblies, Quiver was run iteratively on the previously polished version of the assembly, and results are shown for each PfSPZ assembly. The X-axis is the number of Quiver iterations (1-5); the y-axis is the percent difference from the 3D7 reference genome, which includes both base pair differences (such as single-nucleotide polymorphism and indels) and regions of the 3D7 genome that are not present in the PfSPZ assembly.


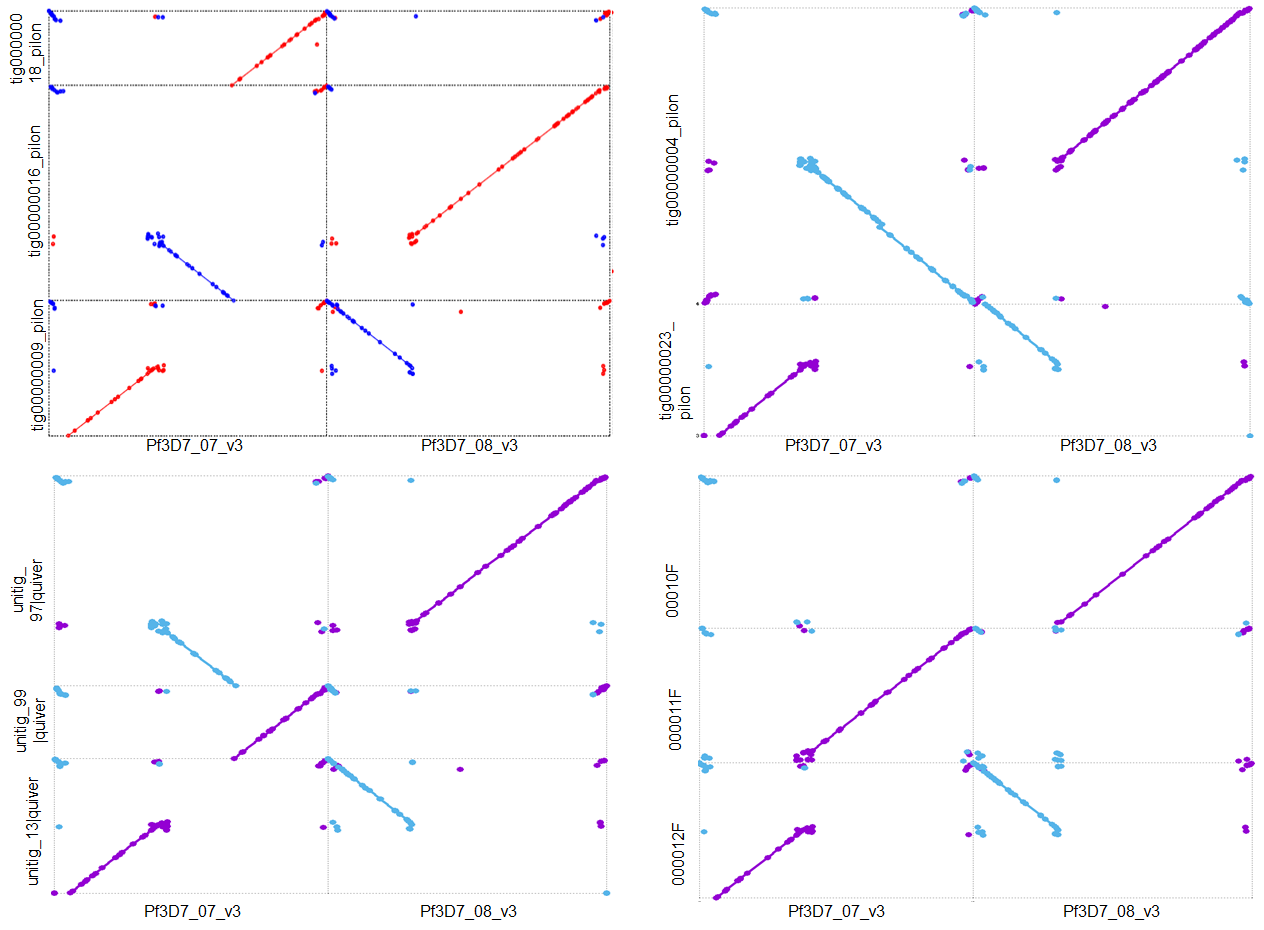


**Fig. 2: A chromosomal** **translocation in NF135.C10, between chromosomes 7 and 8, is supported by multiple assemblers and long read data types.** Mummerplots of NF135.C10 assembly contigs against the 3D7 reference genome show a translocation and inversion of the end of chromosome 8 and a middle section of chromosome 7. The original rearrangement in NF135.C10 Canu Pacbio assembly is shown in the top left. Red alignments indicate an alignment in the same orientation as the 3D7 reference; blue alignments indicate an alignment in the reverse orientation. These are followed (clockwise) by a Canu Pacbio+Nanopore hybrid assembly, an HGAP PacBio assembly, and a Falcon PacBio assembly. Light blue are reverse alignments, purple are alignments in the same orientation as 3D7. While there is variation across assemblers as to the exact chromosome structure, all assembly methods support a rearrangement between the end of chromosome 8 and a middle section of chromosome 7


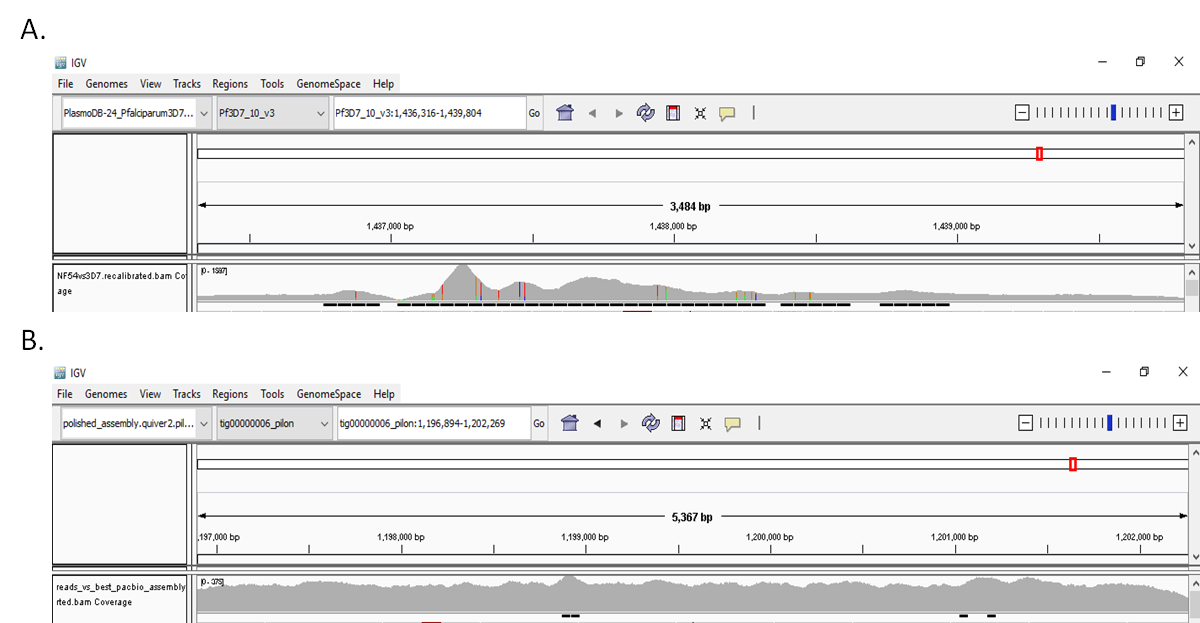


**Fig. S3:** **Illumina read coverage of the repetitive region of liver stage antigen 1 (LSA-1) suggests that the region is longer in NF54 than it is in the 3D7 reference genome.** **A**. NF54 Illumina reads mapped against the LSA-1 locus on chromosome 10 of the 3D7 reference genome visualized in the Integrative Genome Browser (LSA-1 coordinates: 1,436,316 to 1,439,804 bp). **B.** NF54 Illumina reads mapped against the homologous region in the NF54 assembly (tig00000006_pilon, LSA-1 coordinates: 1,196,860 to 1,202,266 bp).

PF3D7_1222600 2299 INNDATITFNNPLNNKNVPYQYLNLKDKKQTFQQYNHIKDRENSYPYMHIKGETQQNGMKLKNDINNHHSQNQLSLSKQENNIYKNNVMD
NF54 2299 INNDATITFNNPLNNKNVPYQYLNLKDKKQTFQQYNHIKDRENSYPYMHIKGETQQNGMKLKNDINNHHSQNQLSLSKQENNIYKNNVMD
7G8 2300 INNDATITFNNPLNNKNVPYQYLNLKDKKQTFQQYNHIKDRENSYPYMHIKGETQQNGMKLKNDINNHHSQNQLSLSKQENNIYKNNVMD
NF166 2300 INNDATITFNNPLNNKNVPYQYLNLKDKKQTFQQYNHIKDRENSYPYMHIKGETQQNGMKLKNDINNHHSQNQLSLSKQENNIYKNNVMD
**NF135.C10** 2303 INNDATITFNNPLNNKNVPYQYLNLKDKKQTFQQYNHIKDRENSYPYMHIKGETQQNGMKLKNDINNHHSQNQLSLSKKENNIYKNNVMD
PfGA01_12002730 2294 INNDATITFNNPLNNKNVPYQYLNLKDKKQTFQQYNHIKDRENSYPYMHIKGETQQNGMKLKNDINNHHSQNQLSLSKQENNIYKNNVMD
PfGN01_12002860 2305 INNDATITFNNPLNNKNVPYQYLNLKDKKQTFQQYNHIKDRENSYPYMHIKGETQQNGMKLKNDINNHHSQNQLSLSKQENNIYKNNVMD
PfCD01_12002750 2304 INNDATITFNNPLNNKNVPYQYLNLKDKKQTFQQYNHIKDRENSYPYMHIKGETQQNGMKLKNDINNHHSQNQLSLSKQENNIYKNNVMD
PfSN01_12002820 2321 INNDATITFNNPLNNKNVPYQYLNLKDKKQTFQQYNHIKDRENSYPYMHIKGETQQNGMKLKNDINNHHSQNQLSLSKQENNIYKNNVMD
PfSD01_12002750 2309 INNDATITFNNPLNNKNVPYQYLNLKDKKQTFQQYNHIKDRENSYPYMHIKGETQQNGMKLKNDINNHHSQNQLSLSKQENNIYKNNVMD
PfML01_12002780 2305 INNDATITFNNPLNNKNVPYQYLNLKDKKQTFQQYNHIKDRENSYPYMHIKGETQQNGMKLKNDINNHHSQNQLSLSKQENNIYKNNVMD
PfGB4_120027200 2304 INNDATITFNNPLNNKNVPYQYLNLKDKKQTFQQYNHIKDRENSYPYMHIKGETQQNGMKLKNDINNHHSQNQLSLSKQENNIYKNNVMD
PfKE01_12002760 2300 INNDATITFNNPLNNKNVPYQYLNLKDKKQTFQQYNHIKDRENSYPYMHIKGETQQNGMKLKNDINNHHSQNQLSLSKQENNIYKNNVMD
PfTG01_12002750 2300 INNDATITFNNPLNNKNVPYQYLNLKDKKQTFQQYNHIKDRENSYPYMHIKGETQQNGMKLKNDINNHHSQNQLSLSKQENNIYKNNVMD
PfHB3_120027400 2290 INNDATITFNNPLNNKNVPYQYLNLKDKKQTFQQYNHIKDRENSYPYMHIKGETQQNGMKLKNDINNHHSQNQLSLSKQENNIYKNNVMD
**PfIT_120027700** 2299 INNDATITFNNPLNNKNVPYQYLNLKDKKQTFQQYNHIKDRENSYPYMHIKGETQQNGMKLKNDINNHHSQNQLSLSKKENNIYKNNVMD
**PfDd2_120027000** 2302 INNDATITFNNPLNNKNVPYQYLNLKDKKQTFQQYNHIKDRENSYPYMHIKGETQQNGMKLKNDINNHHSQNQLSLSKKENNIYKNNVMD
**PfKH01_12002900** 2296 INNDATITFNNPLNNKNVPYQYLNLKDKKQTFQQYNHIKDRENSYPYMHIKGETQQNGMKLKNDINNHHSQNQLSLSKKENNIYKNNVMD
**PfKH02_12002760** 2295 INNDATITFNNPLNNKNVPYQYLNLKDKKQTFQQYNHIKDRENSYPYMHIKGETQQNGMKLKNDINNHHSQNQLSLSKKENNIYKNNVMD
PPRFG01_1232200 2286 INNDATITFNNPLNNKNVPYQYLNLKDKKQTFQQYNHIKDRENSYPYMHIKGETQQNGMKLKNDINNHHSQNQLSSSNQENNIYKNNVMD
PRCDC_1221900 2231 IDNDATITFNNLLNNKNVPYQYLNVKEKKQTFQQYNHIKDREISYPYMHIKGETQQNSMKLKNDINNHHSQNQLSLSNQENNIYKNNVMD


PF3D7_1222600 2389 EKLLIQNLKACINESNNNN-NNN------------------------------------------------------------------N
NF54 2389 EKLLIQNLKACINESNNNN-NNN------------------------------------------------------------------N
7G8 2390 EKLLIQNLKACINESNNNN-NNN------------------------------------------------------------------N
NF166 2390 EKLLIQNLKACINESNNNNNNNN------------------------------------------------------------------N
**NF135.C10** 2393 EKLLIQNLKACINESNNNNNNNSSSSSNSNTNGSYKNFITNTNHIRNLDNNSYNANNNFNTQVNSSS*YTPNDNSILNNNNNNNNNNN-N
PfGA01_12002730 2384 EKLLIQNLKACINESNNNN-NNN------------------------------------------------------------------N
PfGN01_12002860 2395 EKLLIQNLKACINESNNNN-NNN------------------------------------------------------------------N
PfCD01_12002750 2394 EKLLIQNLKACINESNNNN-NNN------------------------------------------------------------------N
PfSN01_12002820 2411 EKLLIQNLKACINESNNNN-NNN------------------------------------------------------------------N
PfSD01_12002750 2399 EKLLIQNLKACINESNNNN-NNN------------------------------------------------------------------N
PfML01_12002780 2395 EKLLIQNLKACINESNNNNNNNN------------------------------------------------------------------N
PfGB4_120027200 2394 EKLLIQNLKACINESNNNN-NNN------------------------------------------------------------------N
PfKE01_12002760 2390 EKLLIQNLKACINESNNNN-NNN------------------------------------------------------------------N
PfTG01_12002750 2390 EKLLIQNLKACINESNNNN-NNN------------------------------------------------------------------N
PfHB3_120027400 2380 EKLLIQNLKACINESNNNN-NNN------------------------------------------------------------------N
**PfIT_120027700** 2389 EKLLIQNLKACINESNNNNNNNSSSSSNSNTNGSYKNFITNTNHIRNLDNNSYNANNNFNTQVNSSS*YTPNDNSILNNNNNNNNNNN-N
**PfDd2_120027000** 2392 EKLLIQNLKACINESNNNNNNNSSSSSNSNTNGSYKNFITNTNHIRNLDNNSYNANNNFNTQVNSSS*YTPNDNSILNNNNNNNNNNN-N
**PfKH01_12002900** 2386 EKLLIQNLKACINESNNNNNNNSSSSSNSNTNGSYKNFITNTNHIRNLDNNSYNANNNFNTQVNSSS*YTPNDNSILNNNNNNNNNNN-N
**PfKH02_12002760** 2385 EKLLIQNLKACINESNNNNNNNSSSSSNSNTNGSYKNFITNTNHIRNLDNNSYNANNNFNTQVNSSS*YTPNDNSILNNNNNNNNNNN-N
PPRFG01_1232200 2376 EKLLIQNLKACINESNNNN-NNSSSSSNSNTNGSYKNFITNTNHIRNLDNNSYNANNNFNTQVNSSSEYTPNDNSILNNNNNNNNNNNNN
PRCDC_1221900 2321 EKLLIQNLQACINESNNNN----SSSSNSNTNGSYKNFLTNTNHIRNLDNNSYNANNNFNTQVNSSSEYTPNDNSILNNNNNNN-----N

PF3D7_1222600 2412 YNVQKRIQNNYYHKGGNNRNI*
NF54 2412 YNVQKRIQNNYYHKGGNNRNI*
7G8 2413 YNVQKRIQNNYYHKGGNNRNI*
NF166 2414 YNVQKRIQNNYYHKGGNNRNI*
**NF135.C10** 2481 YNVQKRIQNNYYHKGGNNRNI*
PfGA01_12002730 2407 YNVQKRIQNNYYHKGGNNRNI*
PfGN01_12002860 2418 YNVQKRIQNNYYHKGGNNRNI*
PfCD01_12002750 2417 YNVQKRIQNNYYHKGGNNRNI*
PfSN01_12002820 2434 YNVQKRIQNNYYHKGGNNRNI*
PfSD01_12002750 2422 YNVQKRIQNNYYHKGGNNRNI*
PfML01_12002780 2419 YNVQKRIQNNYYHKGGNNRNI*
PfGB4_120027200 2417 YNVQKRIQNNYYHKGGNNRNI*
PfKE01_12002760 2413 YNVQKRIQNNYYHKGGNNRNI*
PfTG01_12002750 2413 YNVQKRIQNNYYHKGGNNRNI*
PfHB3_120027400 2403 YNVQKRIQNNYYHKGGNNRNI*
**PfIT_120027700** 2477 YNVQKRIQNNYYHKGGNNRNI*
**PfDd2_120027000** 2480 YNVQKRIQNNYYHKGGNNRNI*
**PfKH01_12002900** 2474 YNVQKRIQNNYYHKGGNNRNI*
**PfKH02_12002760** 2473 YNVQKRIQNNYYHKGGNNRNI*
PPRFG01_1232200 2465 YNVQKRIQNNYYHKGGNNRNI*
PRCDC_1221900 2402 YNMQKRIQNNYYHKGGNNRNI*

**Fig. S4: An indel in the 3’ end of AP2-G (PF1222600) appears to be specific to isolates from Southeast Asia (including the heterologous CHMI clone NF135.C10).** The alignment above shows the 3’end of the AP2-G gene. 3D7 and the four PfSPZ clones are shown, followed by the AP2-G sequences from previously published long-read assemblies (reference 32 in main manuscript) and two non-human primate *Plasmodium* species (PPRFG01: *P. praefalciparum*; PRCDC: *P. reichenowi*). Sequences from the latter two groups were pulled from PlasmoDBv41. A premature stop codon (yellow highlighting) in a ~68 amino acid insertion (relative to 3D7) in isolates of known or suspected Southeast Asian origin (bold text) is shown. PfIT, while originally sampled from South America, has previously been shown to have signs of contamination, sharing genetic singles with isolates from Southeast Asia (4).

**
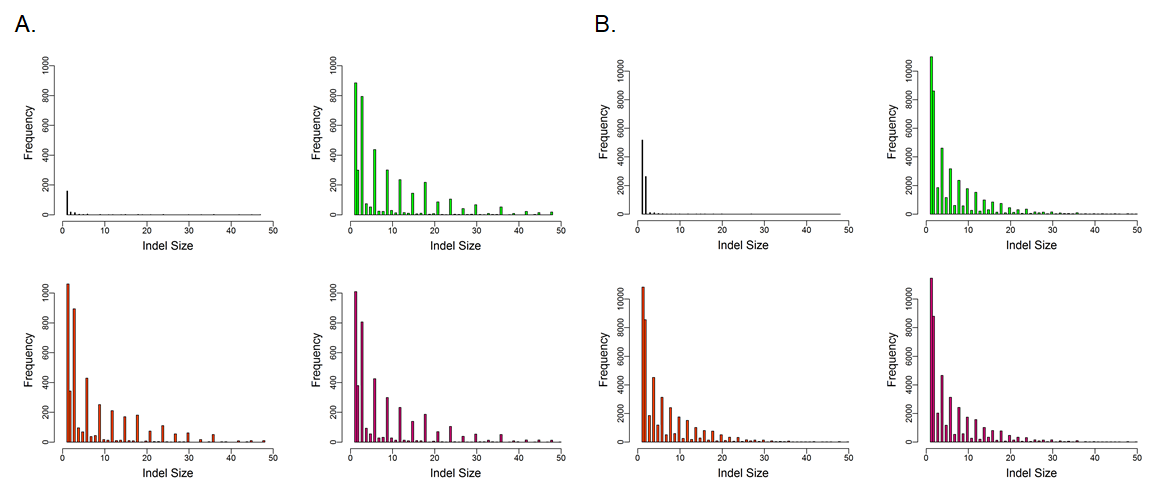
**

**Fig. S5: Distribution of indels.** Small indels (insertions and deletions < 50 bp in size) were identified in each assembly. **A.** Histograms of indels in each assembly in coding regions show that indels tend to be a size in length of multiples-of-three, although some non-multiples-of-three occur (particularly single-bp indels) possibly representing remaining sequencing error). **B.** Histograms of indels from non-coding regions in each assembly show that multiples-of-two indels are more common. NF54 (black), 7G8 (green), NF166.C8 (orange), and NF135.C10 (pink). Small indels with length multiple of three (but not two) base pairs are common in coding regions across the genome, as expected from purifying selection on mutations that disrupt the reading frame, whereas indels in non-coding regions were primarily of lengths multiple of two, from unequal crossover or replication slippage in the regions of dinucleotide repeats common in the *P. falciparum* genome, confirming what has been shown in previous work using short read whole genome sequence data (5).

**
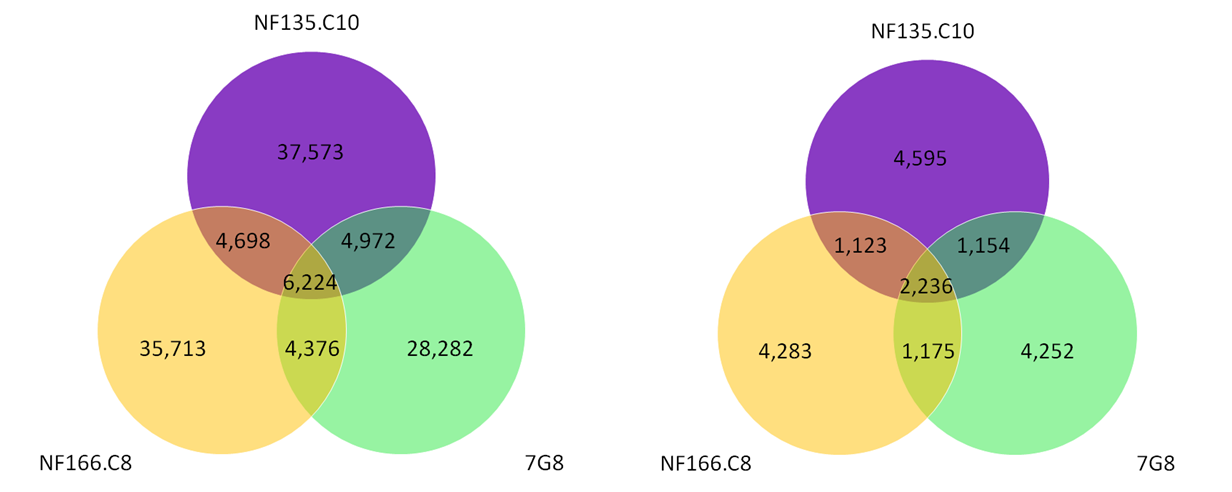
**

**Fig. S6: Shared and unique SNPs between the four PfSPZ strains.** Genome-wide (left) and restricted to non-synonymous SNPs in the core regions of the genome, as defined in main manuscript reference 32 (right). SNPs were detected by aligning each genome to the 3D7 genome with nucmer and nucmer’s show-snps to detect variants. Unique and shared SNPs were then characterized by comparing the positions of SNPs in each heterologous CHMI dataset.


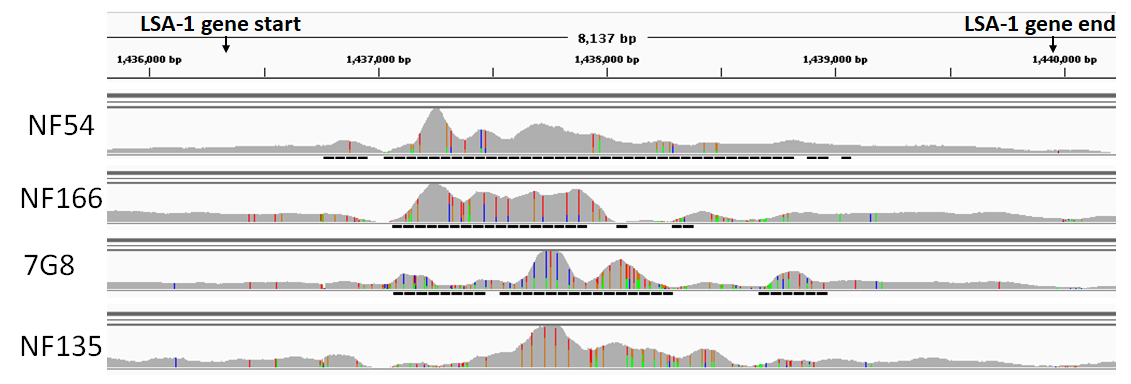


**Fig. S7: The repeat region of LSA-1 is variable in the four PfSPZ strains.** Mapping Illumina reads from each PfSPZ strain against the 3D7 reference revealed a pile-up of reads on chromosome 10 in a region containing liver stage antigen 1 (LSA-1) (visualized here in Integrative Genome Browser; LSA-1 coordinates: 1,436,316 to 1,439,804 bp) as was shown between NF54 and 3D7. The differences in read depth and coverage of this region also reflect variations in the size of the repeat region of LSA-1. While we have confirmed that the lengths of the LSA-1 locus in the three heterologous CHMI strains are also longer than the 3D7 reference genome, note that this figure also reflects the possible 3D7 reference LSA-1 loci error described above.


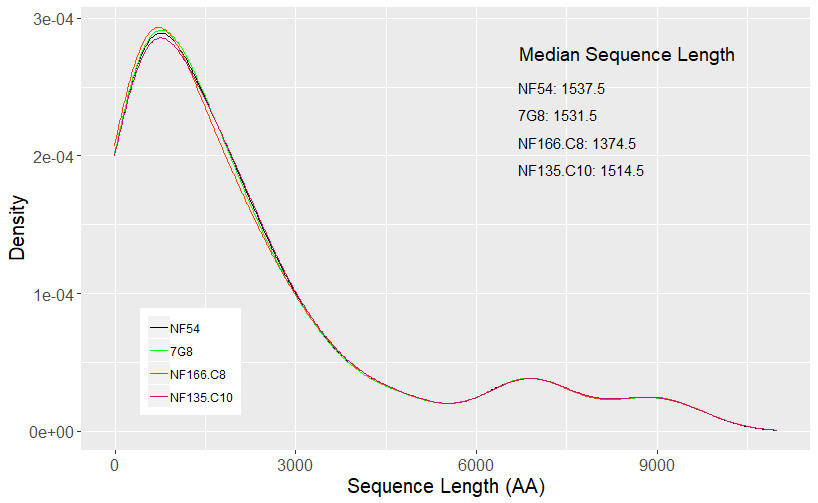


**Fig. S8: Density plots showing the length of amino acid sequences from 42 genes of interest used to predict CD8+ T cell epitopes in NF54 and the three heterologous CHMI strains.** Overall length distributions were very similar across all four PfSPZ strains. However, both the median and average sequence length for NF54 was slightly longer than the other three heterologous CHMI strains.

**Fig. S9: Predicted CD8^+^ T cell epitopes, by position, in selected pre-erythrocytic genes of interest. From top to bottom: PfLISP2, PfLSA3, PfAMA1, & PfTRAP**. Protein domain information based on the 3D7 reference sequence for each gene is found in the first track. The following tracks are epitopes predicted in each gene’s amino acid sequences for NF54, 7G8, NF166.C8, and NF135.C10, respectively. Each box is a sequence that was identified as an epitope, and colors represent the HLA type that identified the epitope.

**
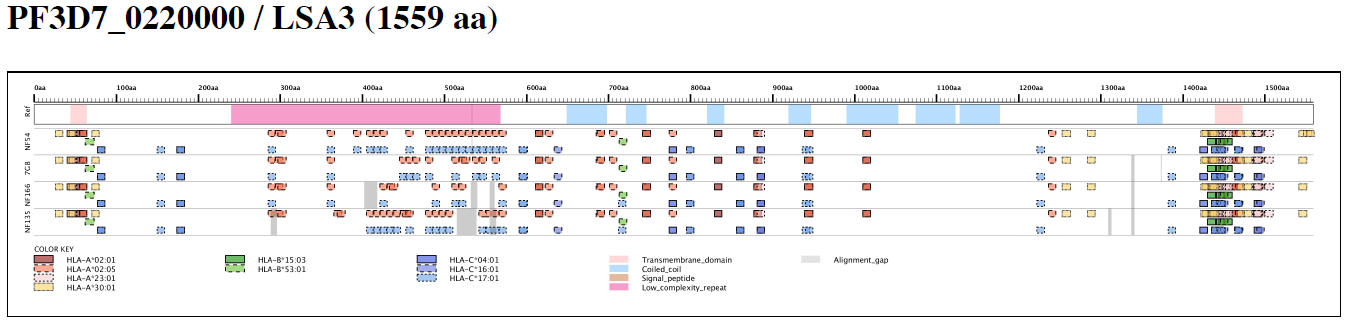
**

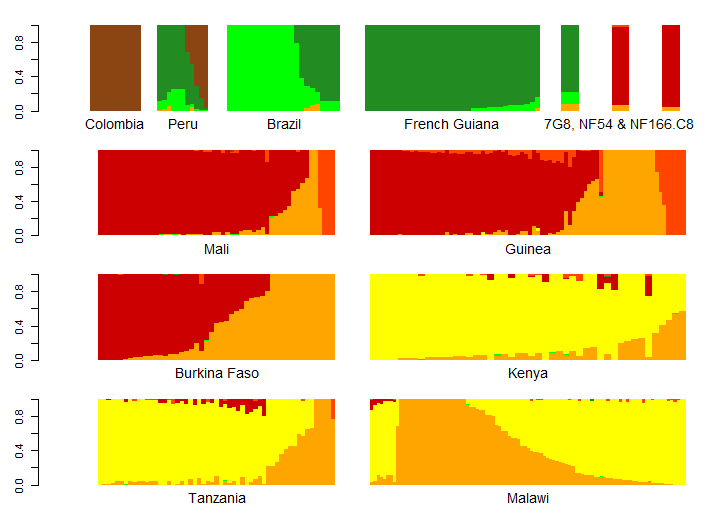


**Fig. S10:** Admixture analysis of South American and African clinical isolates place NF54, 7G8, and NF166.C8 amongst clinical isolates from their respective geographic origins. Admixture analysis was done using 16,802 biallelic variable positions identified in 461 isolates from South America and Africa, along with NF54, NF166.C8, and 7G8, identifying *K*=7 subpopulations. Each color represents one of seven subpopulations; each column of a plot is a sample, with the height of each bar representing the proportion of the genome that was assigned by the model to one of the *K* subpopulations. (Single bars representing 7G8, NF54, and NF166.C8 have been enlarged to aid visualization.)

**Supplemental References**

1. Carneiro MO, Russ C, Ross MG, Gabriel SB, Nusbaum C, DePristo MA. Pacific biosciences sequencing technology for genotyping and variation discovery in human data. BMC Genomics. 2012 Aug 5;13:375.

2. Chin C-S, Alexander DH, Marks P, Klammer AA, Drake J, Heiner C, et al. Nonhybrid, finished microbial genome assemblies from long-read SMRT sequencing data. Nat Meth. 2013 Jun;10(6):563–9.

3. Chin C-S, Peluso P, Sedlazeck FJ, Nattestad M, Concepcion GT, Clum A, et al. Phased diploid genome assembly with single-molecule real-time sequencing. Nat Methods. 2016 Dec;13(12):1050–4.

4. Robson KJH, Walliker D, Creasey A, McBride J, Beale G, Wilson RJM. Cross-contamination of *Plasmodium* cultures. Parasitology Today. 1992 Feb 1;8(2):38–9.

5. Miles A, Iqbal Z, Vauterin P, Pearson R, Campino S, Theron M, et al. Indels, structural variation, and recombination drive genomic diversity in *Plasmodium falciparum*. Genome Res. 2016 Sep 1;26(9):1288–99.
